# What can we learn from 1,000 meta-analyses across 10 different disciplines?

**DOI:** 10.1017/rsm.2025.10035

**Published:** 2025-10-02

**Authors:** Weilun Wu, Jianhua Duan, W. Robert Reed, Elizabeth Tipton

**Affiliations:** 1 Department of Economics and Finance and UCMeta, University of Canterbury, Christchurch, New Zealand; 2 Statistics New Zealand, Christchurch, New Zealand; 3 Statistics for Evidence-Based Policy and Practice Center, Northwestern University, Evanston, IL, USA

**Keywords:** meta-analysis, interdisciplinary comparison, tests for publication bias, clustering, meta-analytic estimators, meta-regression

## Abstract

This study analyzes 1,000 meta-analyses drawn from 10 disciplines—including medicine, psychology, education, biology, and economics—to document and compare methodological practices across fields. We find large differences in the size of meta-analyses, the number of effect sizes per study, and the types of effect sizes used. Disciplines also vary in their use of unpublished studies, the frequency and type of tests for publication bias, and whether they attempt to correct for it. Notably, many meta-analyses include multiple effect sizes from the same study, yet fail to account for statistical dependence in their analyses. We document the limited use of advanced methods—such as multilevel models and cluster-adjusted standard errors—that can accommodate dependent data structures. Correlations are frequently used as effect sizes in some disciplines, yet researchers often fail to address the methodological issues this introduces, including biased weighting and misleading tests for publication bias. We also find that meta-regression is underutilized, even when sample sizes are large enough to support it. This work serves as a resource for researchers conducting their first meta-analyses, as a benchmark for researchers designing simulation experiments, and as a reference for applied meta-analysts aiming to improve their methodological practices.

## Highlights

### What is already known?

- Meta-analysis is widely used across disciplines, but practices vary considerably.
- Prior reviews of meta-analytic methods have often focused on single disciplines or limited samples.

### What is new?

- This study analyzes 1,000 meta-analyses from 10 disciplines in medicine, science, and the social sciences, revealing differences in study size, effect sizes, estimators, heterogeneity, and publication bias practices.
- It highlights four key areas for improvement: addressing data dependence, correcting for publication bias, using meta-regression, and properly handling correlation-based effect sizes.

### Potential impact for RSM readers

- Offers a rare, cross-disciplinary overview of meta-analytic practices.
- Serves as a guide for first-time meta-analysts, a benchmark for simulation design, and a reference for improving applied practice.

## 1 Introduction

Meta-analysis has become an increasingly important tool for synthesizing empirical findings across a wide spectrum of disciplines. This study contributes to the understanding of meta-analytic practices by describing and analyzing 1,000 meta-analyses across 10 different disciplines spanning medicine, science, and the social sciences. Our primary objective is to assess current meta-analytic practices across these disciplines. Specifically, we examine the scope and types of meta-analyses conducted, as well as the common methods used to analyze meta-analytic data. We code key aspects—such as the number of studies, the number of estimates per study, and the degree of effect heterogeneity—and compare these across disciplines. For example, we find that in medicine, meta-analyses are often small (3–5 studies), while in the social sciences they are often large (> 50 studies). We expect this descriptive information to be particularly useful to statistical and methodological researchers developing new methods and conducting simulation studies.

A primary focus of this paper is to examine the methodological approaches used across different fields and to assess their adequacy in light of established statistical and methodological guidelines. In some instances, we find that current practices diverge from recommended guidelines, with analysts employing methods that are inappropriate given the nature of the data and prevailing best practices. For example, in some fields, dependent effect sizes are routinely analyzed as if they were independent. By identifying such mismatches, we aim to highlight areas where methodological practice can be strengthened and to encourage improvements across disciplines.

To achieve these objectives, we analyzed the first 100 meta-analyses published in November 2021 within each of the following 10 disciplines: Anatomy and Physiology; Biology; Business and Economics; Education; Engineering; Environmental Sciences; Medicine; Pharmacy, Therapeutics and Pharmacology; Psychology; and Public Health. While we aimed for a robust and representative sample within the constraints of available resources, our selection of the first 100 meta-analyses within a specific timeframe provides a snapshot of practices during that period. To prevent oversampling from any single journal, we capped the number of meta-analyses drawn from each journal at 10.

Our study describes a range of key characteristics, such as how many studies and estimates were included in meta-analyses and the average number of estimates per study. We record whether unpublished studies were incorporated, the type(s) of effect sizes analyzed, the estimator(s) used (e.g., fixed effects, random effects), and how heterogeneity was reported and quantified. We investigate whether publication bias was assessed, the specific methods employed for that purpose, and whether publication bias was found. We also identify the types of software used to conduct the analyses (e.g., R, Stata, CMA). Based on this analysis, we call attention to four areas of practice that could benefit from improvement.

This study proceeds as follows. Section 2 outlines the search process that selected the 1,000 meta-analyses for our study. Section 3 presents descriptive statistics for the meta-analyses from the 10 disciplines in our study. Section 4 provides evidence and recommendations for improving four key areas of meta-analytic practice. Section 5 concludes.

## 2 Meta-analysis search process

The initial research for this study originated from a blog project exploring disciplinary differences in how meta-analyses are conducted.^1^ That preliminary effort sampled 20 meta-analyses from 18 different disciplines. Building on this work, we expanded the scope to include 100 meta-analyses from each of 10 disciplines. We selected this sample size to ensure a robust and representative set of studies within each field, and chose 10 disciplines to enable meaningful cross-disciplinary comparisons. While a larger sample—either in terms of disciplines or meta-analyses per discipline—would have been desirable, practical constraints on time and resources limited the scope of data collection and analysis.

To identify relevant studies, we used the proprietary discovery service Summon^2^, available through the university library of the first three authors. Summon is widely adopted by academic, research, and public libraries worldwide. It enables unified searches across multiple databases (e.g., Scopus, JSTOR, PubMed) through a single interface and is particularly valuable for our purposes because it categorizes search results by research discipline. The version we used, Summon 2.0, includes 61 discipline categories spanning the natural sciences, social sciences, applied sciences, and more. For each discipline, we searched using the keyword “meta-analysis,” filtering results to include only peer-reviewed journal articles.

Our aim was to identify the 10 disciplines with the highest number of meta-analyses. Here, we acknowledge a degree of disciplinary bias: the home discipline of three authors is Economics, which ranked 13th among the 61 disciplines. To include Economics in our analysis, we combined it with Business (ranked 17th), yielding a sufficiently large set of studies to place the combined category within the top 10. The final list of disciplines in our sample was: Anatomy and Physiology; Biology; Business and Economics; Education; Engineering; Environmental Sciences; Medicine; Pharmacy, Therapeutics and Pharmacology; Psychology; and Public Health.

After selecting the 10 disciplines, we returned to Summon and collected the first 100 meta-analyses published in November 2021 for each field, based on the order in which they appeared. If a discipline did not yield 100 meta-analyses for that month, we extended the search to earlier periods. To avoid overrepresentation from any single journal, we capped the number of meta-analyses per journal at 10. Duplicate entries were removed and replaced with studies published as close as possible to November 2021. The final list of journals for each discipline is provided in an Excel spreadsheet at https://osf.io/6dgpn. Coding was carried out by multiple teams, with each meta-analysis coded by at least two researchers (and often more), and subsequently reviewed and recoded multiple times by the authors over a multi-year period.

## 3 A description of 1,000 meta-analyses across 10 disciplines

### 3.1 Number of studies/number of estimates

Even a cursory inspection of meta-analyses reveals substantial variation in the number of included estimates and studies. Complicating our task of recording this information was the fact that many studies report multiple meta-analyses—typically for different subsamples—rather than a single, combined meta-analysis. In such cases, we selected the largest meta-analysis for inclusion in our study.

Table 1 reports the median and mean values of number of studies and number of estimates for each of the 10 disciplines. The disciplines are arranged in ascending order, with the disciplines having the smallest median number of studies at the top of the table. Overall, the median (mean) number of studies in a meta-analysis for the full sample is 21 (38.0), and the median (mean) number of estimates is 28 (175.7). However, there are substantial differences across disciplines.

**Table 1:** Number of studies and estimates in meta-analyses

	No. of studies		No. of estimates	
Discipline	Median	Mean	Median	Mean
Pharmacy, Therapeutics and Pharmacology	11	16.3	13	23.0
Anatomy and Physiology	14	21.8	19.5	31.2
Public Health	15	30.4	17.5	182.1
Engineering	15.5	33.3	20.5	134.0
Medicine	16	20.8	17.5	26.1
Biology	21	36.9	25.5	297.7
Environmental Sciences	22.5	39.6	63.5	184.0
Education	28.5	49.6	41.5	205.8
Psychology	32	58.7	62	136.7
Business and Economics	53.5	72.4	163.5	536.7

**Overall**	**21**	**38.0**	**28**	**175.7**

*Note*: The data in the table are based on 100 meta-analyses for each discipline. A more detailed summary is provided in Figures 1 and 2.

Pharmacy, Therapeutics and Pharmacology is characterized by the smallest number of studies, with a median (mean) number of studies of 11 (16.3), and a median (mean) number of estimates of 13 (23.0). Meta-analyses in Business and Economics tend to be the largest, with median (mean) values of 53.5 (72.4) and 163.5 (536.7), respectively. There are also substantial differences within disciplines. For example, the minimum and maximum number of studies for meta-analyses in Pharmacy, Therapeutics and Pharmacology are 2 and 81. In Business and Economics, the minimum and maximum studies are 5 and 613.

**Figure 1:**
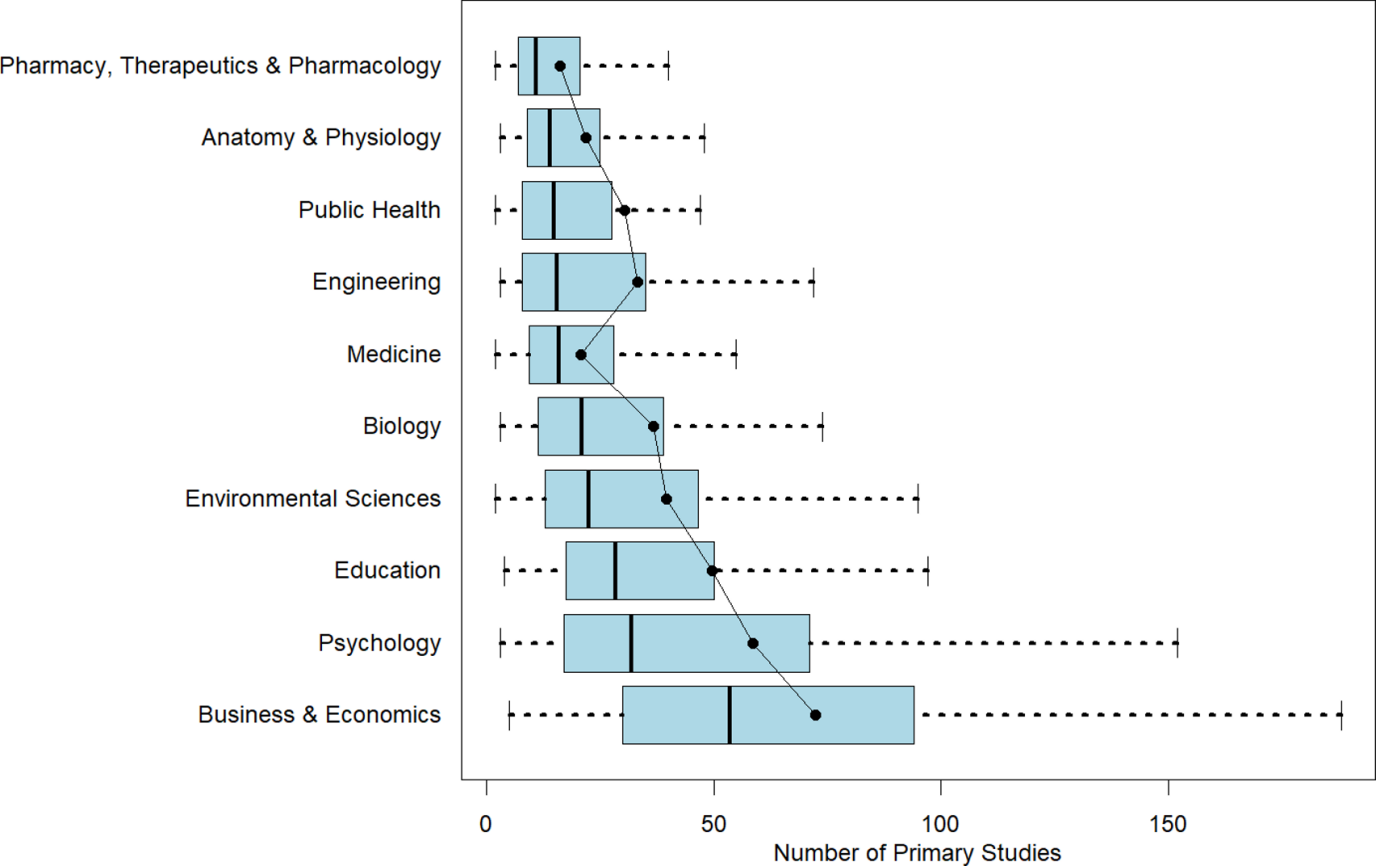
Number of studies in a meta-analysis by discipline. *Note*: Figure 1 reports boxplots (without outliers) for each of the 10 disciplines with respect to the number of studies. Superimposed on the boxplots are the average number of studies per meta-analysis. The figure is arranged with the smallest median number of studies per meta-analysis at the top of the figure to the largest median number of studies at the bottom of the figure.

**Figure 2:**
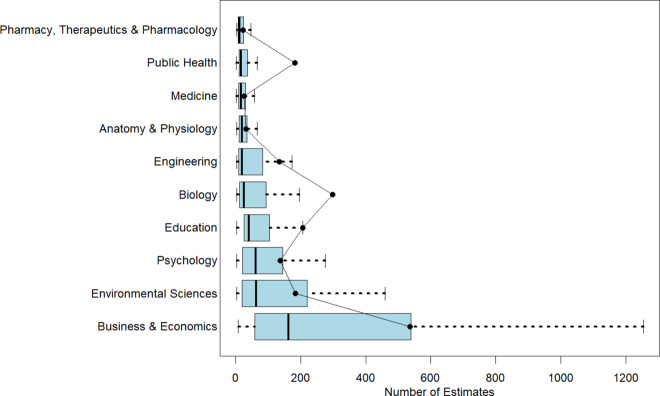
Number of estimates in a meta-analysis by discipline. *Note*: Figure 2 reports boxplots (without outliers) for each of the 10 disciplines with respect to the number of estimates. Superimposed on the boxplots are the average number of studies per meta-analysis. The figure is arranged with the smallest median number of estimates per meta-analysis at the top of the figure to the largest median number of estimates at the bottom of the figure.

This heterogeneity in number of studies and number of estimates is illustrated in Figures 1 and 2. The first figure reports boxplots (without outliers) for each of the 10 disciplines with respect to the number of studies. Superimposed on the boxplots are the average number of studies per meta-analysis. The figure is arranged with the smallest median number of studies per meta-analysis at the top of the figure to largest median number of studies at the bottom of the figure. The second figure does the exact same thing, except for estimates per meta-analysis.

Within-discipline heterogeneity is generally increasing in median value. Pharmacy, Therapeutics and Pharmacology; Anatomy and Physiology; Public Health; and Medicine have least heterogeneity in both number of studies and number of estimates, and also have the lowest or among the lowest median values of studies and estimates. On the other end, Business and Economics has the greatest heterogeneity in numbers of studies and estimates, and largest median values as well.

One insight from this analysis is that it highlights the difficulty of identifying a “typical” meta-analysis size within any given discipline. Fields such as Pharmacy, Therapeutics and Pharmacology; Anatomy and Physiology; Public Health; and Medicine tend to exhibit greater consistency in the number of included studies and estimates. In contrast, Psychology and Business and Economics show much wider variability. For novice meta-analysts or manuscript reviewers working in these latter fields, it can be difficult to judge what constitutes a normative meta-analysis size.

A further benefit of the information in Table 1 and Figures 1 and 2 is its value for informing the design of simulation studies. Researchers conducting simulations to evaluate meta-analytic methods often need to make assumptions about the size and structure of typical datasets. By documenting how these characteristics vary across disciplines, our findings allow researchers to construct simulated datasets that better reflect real-world meta-analytic conditions. This, in turn, enhances the realism, relevance, and generalizability of simulation-based evaluations.

### 3.2 Number of estimates per study

A related characteristic is the number of estimates per study. While the total number of studies and estimates captures overall meta-analysis size, estimates per study draw attention to dependence among observations. For example, the two most common estimators in meta-analysis—fixed effects and random effects—assume that estimated effects are independent, typically reflected in the assumption that each study only has one estimate. If the assumption of independence is violated, it undermines estimator efficiency and compromises the validity of statistical inference. In this section, we investigate whether one estimate per study accurately represents the data structure of most meta-analyses.

Table 2 reports the median and mean number of estimates per study for each discipline, sorted in ascending order of the median. In disciplines such as Medicine; Pharmacy, Therapeutics and Pharmacology; Anatomy and Physiology; Public Health; Biology; and Engineering, the median rounds to 1.0 (to one decimal place).^3^ At the opposite end, disciplines like Environmental Sciences and Business and Economics have median values above 2.

**Table 2:** Number of estimates per study in meta-analyses

	Median estimates/study	Mean estimates/study	% MAs 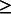 one estimate/study
Discipline	1	2	3
Medicine	1.0	1.2	33%
Pharmacy, Therapeutics and Pharmacology	1.0	1.4	39%
Anatomy and Physiology	1.0	1.6	46%
Public Health	1.0	2.0	47%
Biology	1.0	2.9	48%
Engineering	1.0	3.0	50%
Psychology	1.2	2.1	77%
Education	1.3	2.3	69%
Environmental Sciences	2.0	6.1	78%
Business and Economics	3.1	8.1	84%

**Overall**	**1.1**	**3.1**	**57%**

*Note*: The data in the table are based on 100 meta-analyses for each discipline, so the percentages in Column 3 also provide the counts (e.g., 33% = 33 meta-analyses in the given discipline). As noted, the numbers in the table report median values. A more detailed summary is provided in Figure 3.

These values might suggest that most meta-analyses conform to the one-estimate-per-study assumption. However, this conclusion would be reductive and not fully accurate. The third column of Table 2 shows the percentage of meta-analyses in each discipline that include more than one estimate per study. This reveals a more nuanced picture.

For instance, although Engineering has a median of 1.0, approximately half of its meta-analyses include more than one estimate per study. In Environmental Sciences, over 75% do so; in Business and Economics, the figure exceeds 80%. Across the entire sample of 1,000 meta-analyses, 57% contain more than one estimate per study.

**Figure 3:**
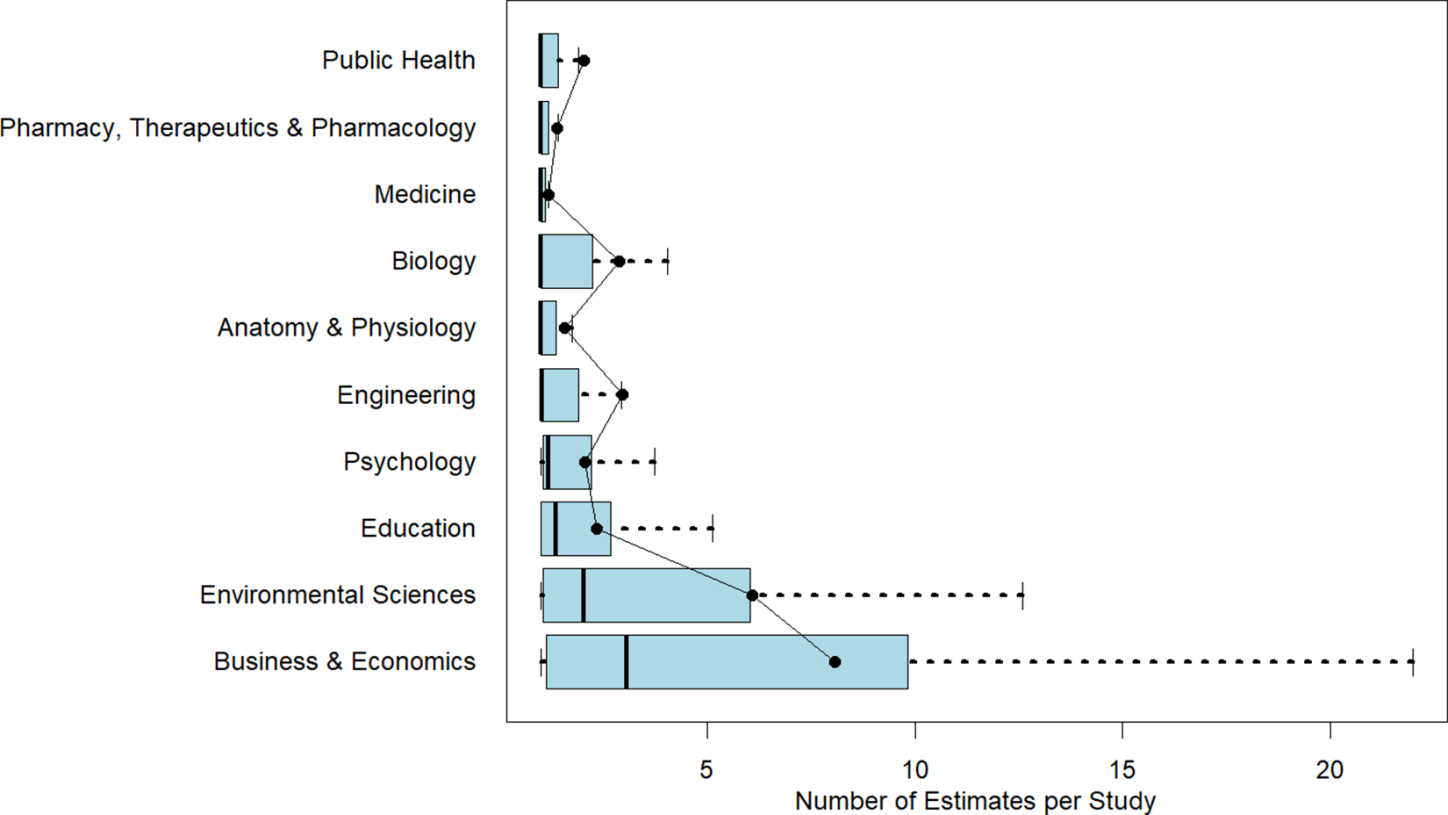
Number of estimates per study in a meta-analysis by discipline. *Note*: Figure 3 reports boxplots (without outliers) for each of the 10 disciplines with respect to the number of estimates per study. Superimposed on the boxplots are the average number of estimates per study. The figure is arranged with the smallest median number of estimates per study at the top of the figure to the largest median number of estimates per study at the bottom of the figure.

Figure 3 presents boxplots showing within-discipline heterogeneity in estimates per study, again sorted by median values. Mean values are superimposed for reference. As seen in earlier figures, heterogeneity increases with the median, further highlighting the variability in practice. Clearly, the one-estimate-per-study assumption may be valid for many meta-analyses, but is inappropriate for many others. We discuss the implications of this for estimator efficiency and inference below.

### 3.3 Inclusion of unpublished studies

Including unpublished studies (a.k.a. “grey literature”) in meta-analyses is a topic of ongoing debate. Proponents argue that its inclusion helps reduce publication bias, as unpublished studies are less likely to report statistically significant or “positive” results. Excluding these sources can, therefore, lead to inflated effect size estimates and distorted conclusions. Grey literature also increases the total number of studies in a meta-analysis, improving statistical power and enabling more precise estimates. In some cases, it may offer more up-to-date findings, particularly in rapidly evolving fields where peer-reviewed publication lags behind current research. Moreover, by capturing a broader array of study designs and contexts, grey literature can enhance the generalizability of results.

On the other hand, grey literature is often not peer-reviewed and may suffer from lower methodological rigor. These sources can be difficult to locate systematically, and the lack of standardization complicates reproducibility. There is also the risk of double-counting results if preliminary findings from grey literature are later published in journal articles. Additionally, identifying, screening, and coding grey literature increases the time and resource demands of a meta-analysis. Prominent guidelines recommend that meta-analyses include grey literature, provided that a careful assessment of research quality is conducted.^1^ ^–^ ^3^An important topic for future discussion is how AI might influence the consensus on including unpublished studies, given its potential to generate large numbers of fake studies.

Table 3 reports both relatively low inclusion rates of unpublished studies in meta-analyses and notable differences across disciplines. In fields such as Environmental Sciences, Medicine, Anatomy and Physiology, Pharmacy, Therapeutics and Pharmacology, and Biology, the inclusion of unpublished studies is relatively uncommon—fewer than one in five meta-analyses incorporate them. In contrast, approximately two-thirds of meta-analyses in Business and Economics and Psychology include unpublished studies. Overall, only about one-third (31%) of the meta-analyses in our full sample included unpublished studies. This highlights a gap between practice and guideline-based recommendations.

**Table 3:** Percent of meta-analyses including unpublished primary studies

Discipline	Percent using unpublished studies
Environmental Sciences	9%
Medicine	14%
Anatomy and Physiology	15%
Pharmacy, Therapeutics and Pharmacology	15%
Biology	18%
Engineering	21%
Public Health	29%
Education	56%
Business and Economics	64%
Psychology	68%

**Overall**	**31%**

*Note*: The data in the table are based on 100 meta-analyses for each discipline, so the percentages in the table also provide the counts (e.g., 9% = 9 meta-analyses in the given discipline).

### 3.4 Effect sizes

Another dimension that varies widely across disciplines is effect sizes. These are the variables that appear as dependent variables in meta-analyses. For the purposes of classification, we categorized effect sizes into the following groups: Ratios, which include measures such as odds ratios, hazard ratios, and risk ratios; Mean Differences (“Mean Diff”), of which the most common measures are Cohen’s d and Hedges’ g; Prevalence, where the effect size is a percentage or frequency; Correlations (“Corr”), representing correlations and partial correlation coefficients; Fisher’s z; and Other, the most common of which are regression coefficients.^4^ We classified effect sizes based on the metric actually used in the analysis. For example, if all primary studies in a meta-analysis reported correlations, but these were transformed to Fisher’s *z* values for estimation (and then converted back to correlations for reporting), the effect size was classified as Fisher’s *z*.

**Figure 4:**
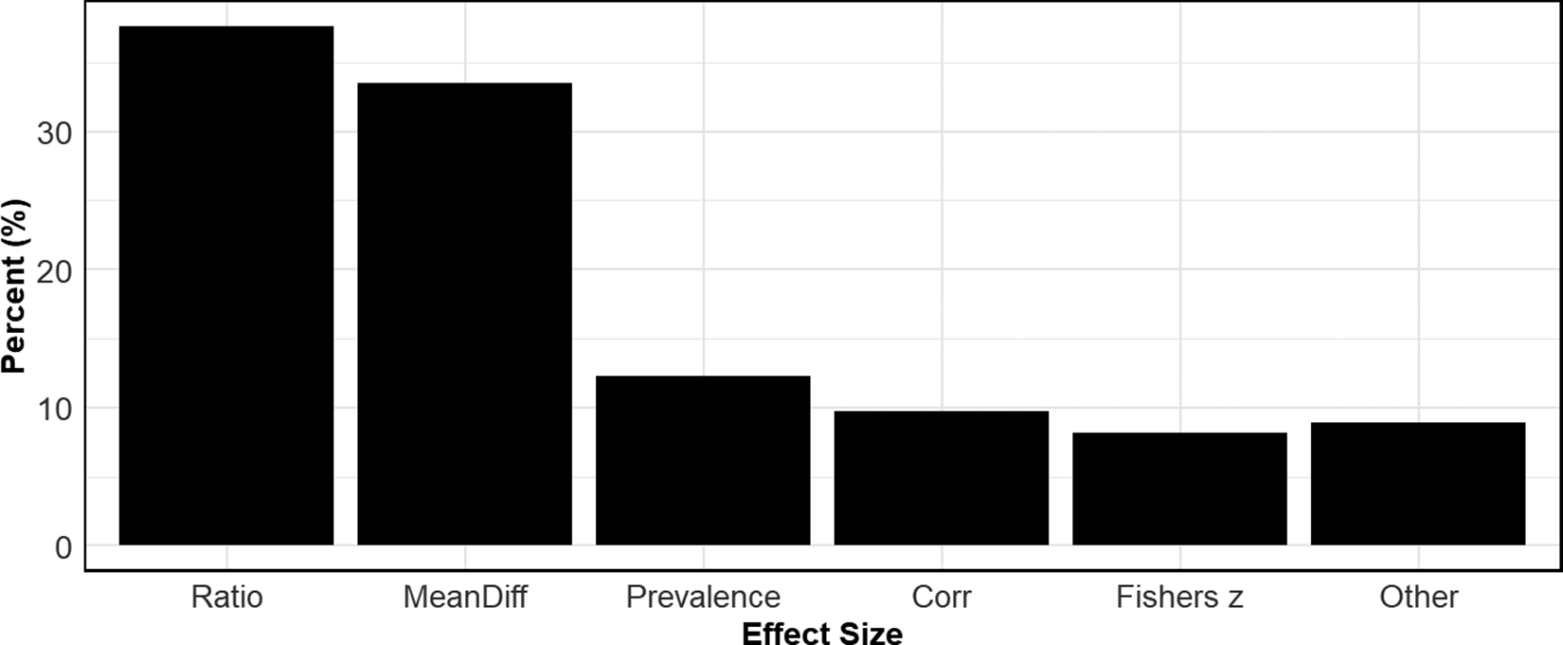
Usage rates of different effect sizes. *Note*: Figure 4 reports aggregate usage rates of different effect sizes across all disciplines. “Ratio” includes odds ratios, hazard ratios, and risk ratios. “Mean Diff” includes Cohen’s *d* and Hedge’s *g*. “Prevalence” uses a frequency or count to measure an outcome. “Corr” stands for correlation and includes partial correlation coefficients.

Figure 4 presents a bar chart that reports overall usage rates of the different types of effect sizes across all disciplines. The most common are ratio measures, with a little over a third of all meta-analyses using some form of a ratio variable to measure effect size. Closely following are the mean difference measures. Beyond these two effect sizes, the other categories are roughly used in equal proportions. Note that the sum of the usage rates exceeds 100% because some meta-analyses use more than one effect size.

Table 4 provides further detail about effect size usage rates by discipline. Disciplines such as Biology; Medicine; and Pharmacy, Therapeutics and Pharmacology most frequently use Ratio-based effect sizes (e.g., odds ratios, hazard ratios, and risk ratios). This could be due to the nature of the data analyzed in these fields, where outcomes are commonly binary. In contrast, fields like Anatomy and Physiology, Education, and Psychology show a stronger preference for Mean Difference measures such as Cohen’s d and Hedges’ g. In these disciplines, it is usual for the outcomes to be continuous and the treatments/interventions to be binary. In Business and Economics, the most frequently used effect size is a correlation. This stems from the fact that it is common in that discipline to combine estimates from regression models that use different outcome and treatment variables. To make the estimates comparable across studies, meta-analyses transform the regression coefficients to partial correlation coefficients (PCCs). Lastly, we note that Fisher’s *z* finds it most frequent use in Business and Economics, Education, and Psychology.

**Table 4:** Prevalence of different effect sizes

Discipline	Ratio	Mean diff.	Prevalence	Corr.	Fishers *z*	Other
Anatomy and Physiology	37%	51%	8%	2%	4%	2%
Biology	64%	25%	16%	1%	3%	5%
Business and Economics	8%	11%	0%	46%	21%	19%
Education	15%	56%	2%	13%	16%	4%
Engineering	44%	36%	11%	6%	3%	13%
Environmental Sciences	44%	23%	9%	5%	4%	24%
Medicine	56%	20%	34%	0%	3%	4%
Pharmacy, Therapeutics and Pharmacology	54%	40%	18%	0%	2%	1%
Psychology	8%	43%	5%	23%	22%	5%
Public Health	47%	31%	21%	3%	2%	8%

**Overall**	**38%**	**34%**	**12%**	**10%**	**8%**	**9%**

*Note*: The data in the table are based on 100 meta-analyses for each discipline, so the percentages in the table also provide the counts (e.g., 37% = 37 meta-analyses in the given discipline). Note that the sum of the percentages (counts) across each row are greater than 100% (100 meta-analyses) because studies can use more than one effect size.

As we discuss below, some effect sizes, particularly correlations/PCCs, have the problem that the standard error of the estimated effect size is a function of the effect size. When inverse-variance weights are used, this introduces bias in estimating overall mean effects and testing for publication bias. The two most common ways to remove this bias are to transform the effect size (e.g., transforming the correlation into Fisher’s *z*), or to use weights other than inverse-variance (e.g., N-weights).

### 3.5 Estimators

**Figure 5:**
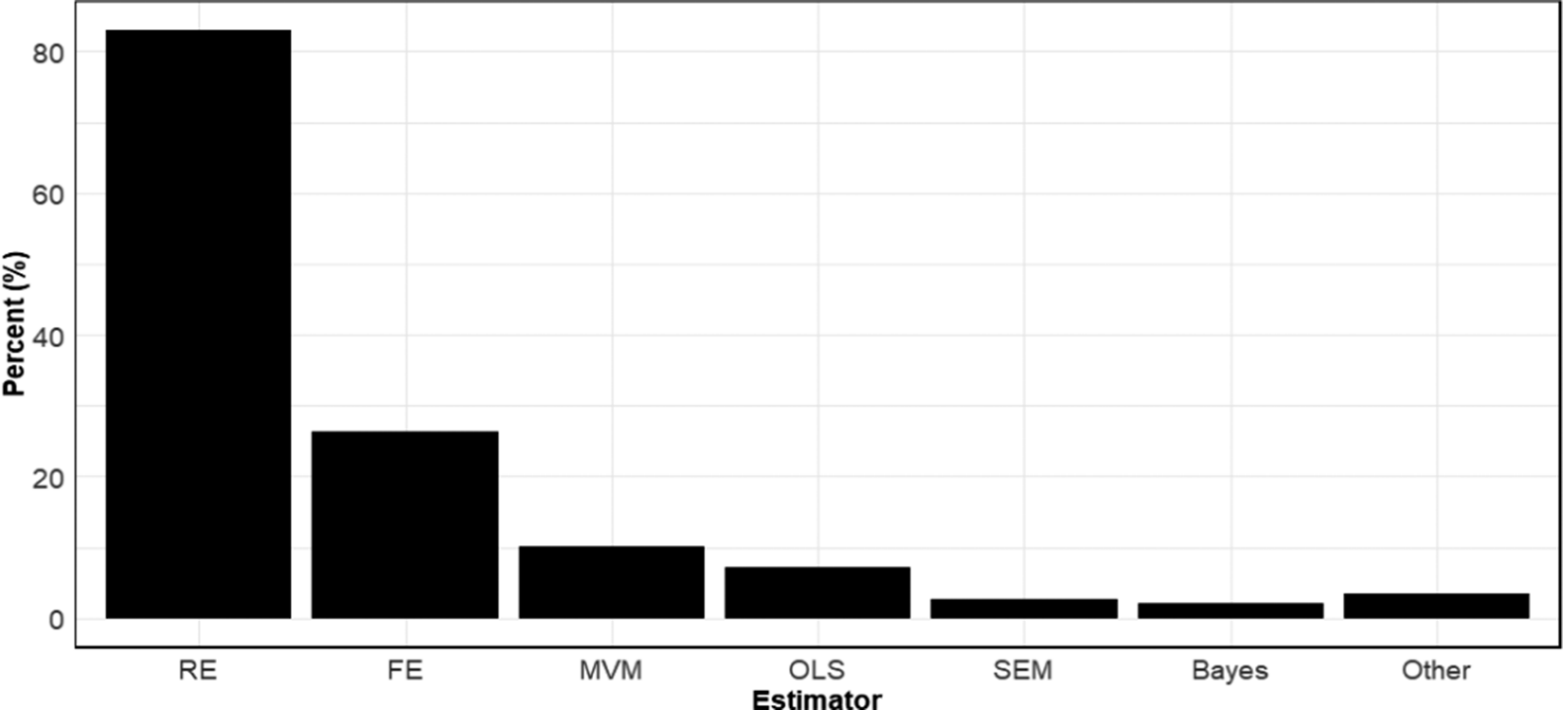
Usage rates of different meta-analytic estimators. *Note*: Figure 5 reports aggregate usage rates of different meta-analytic estimators across all disciplines. RE, random effects; FE, fixed effects; MVM, multi-level model; OLS, ordinary least squares; SEM, structural equation modelling; Bayes, Bayesian estimation.

Figure 5 reports the rates at which different meta-analytic estimators are used. We categorized estimators into seven types: random effects, fixed effects, multivariate/multilevel models (“MVM”), OLS, structural equation models (“SEM”), Bayesian estimators, and Other. While the category MVM includes both multivariate and multilevel models, virtually all the multivariate/multilevel models in our sample were multilevel (three-level) models, which is why we combined them.

Perhaps not surprisingly, random effects is far and away the most used meta-analytic estimator. Over 8 out of 10 meta-analyses in our sample used random effects. Fixed effects was a distant second in terms of frequency of use with approximately 1 in 4 meta-analyses using this estimator. All other estimators were used relatively infrequently. As before, we note that the sum of the usage rates within disciplines exceeds 100% because many meta-analyses employed more than one estimator.

Detailed usage rates by discipline are reported in Table 5. Noteworthy here is that in most disciplines, the only two estimators that matter are random effects and fixed effects. For example, among meta-analyses in Pharmacy, Therapeutics and Pharmacology, 96% used random effects, and 37% used fixed effects. Outside of these, virtually no other estimators were used. Though not as extreme, a similar practice was followed in Anatomy and Physiology, Medicine, and Public Health. In this respect, Business and Economics stands out for its diverse usage of estimators. While random effects is the most used estimator in that discipline, substantial percentages of meta-analyses also use MVM, OLS, SEM, Bayesian methods (often Bayesian model averaging), and others.

**Table 5:** Prevalence of different estimators

Discipline	RE	FE	MVM	OLS	SEM	Bayes	Other
Anatomy and Physiology	91%	32%	5%	2%	0%	2%	1%
Biology	83%	22%	16%	5%	1%	2%	1%
Business and Economics	68%	26%	24%	21%	19%	12%	16%
Education	88%	11%	11%	2%	0%	0%	3%
Engineering	78%	30%	1%	13%	1%	1%	3%
Environmental Sciences	64%	23%	16%	15%	1%	1%	4%
Medicine	87%	34%	4%	4%	0%	1%	1%
Pharmacy, Therapeutics and Pharmacology	96%	37%	1%	0%	0%	0%	3%
Psychology	83%	15%	20%	5%	5%	1%	0%
Public Health	92%	34%	4%	5%	0%	0%	2%

**Overall**	**83%**	**26%**	**10%**	**7%**	**3%**	**2%**	**3%**

*Note*: The data in the table are based on 100 meta-analyses for each discipline, so the percentages in the table also provide the counts (e.g., 92% = 92 meta-analyses in the given discipline). Note that the sum of the percentages (counts) across each row are greater than 100% (100 meta-analyses) because studies can use more than one estimator. RE, random effects; FE, fixed effects; MVM, multivariate/multilevel models; OLS, ordinary least squares; SEM, structural equation models; Bayes, Bayesian estimation.

Table 6 does a deeper dive into the use of random effects and fixed effects estimators. Specifically, it tracks how often a meta-analysis uses only random effects or only fixed effects. We see that rarely is fixed effects the only estimator used by meta-analyses. In contrast, random effects often are. This is because fixed effects is frequently used to provide an initial analysis of the data before it is subsequently rejected in favor of the random effects model. In other words, even though it is the second most widely used estimator, this does not seem to reflect confidence that it is often viewed as best suited for analyzing the data.

**Table 6:** Closer comparison of random effects and fixed effects

Discipline	Only RE	Only FE
Anatomy and Physiology	60%	5%
Biology	57%	0%
Business and Economics	25%	1%
Education	74%	0%
Engineering	50%	4%
Environmental Sciences	42%	1%
Medicine	59%	6%
Pharmacy, Therapeutics and Pharmacology	60%	3%
Psychology	57%	0%
Public Health	56%	1%

**Overall**	**54%**	**2%**

*Note*: The data in the table are based on 100 meta-analyses for each discipline, so the percentages in the table also provide the counts (e.g., 64% = 64 meta-analyses in the given discipline). Only RE reports how many meta-analyses use only one estimator and that estimator is random effects. Only FE reports how many meta-analyses use only one estimator and that estimator is fixed effects.

### 3.6 Heterogeneity

One reason for conducting meta-analyses is to combine studies that examine the effect of a common treatment or intervention, with the goal of obtaining a more precise estimate of that effect. A meaningful and more accurate estimate is most likely when the studies being pooled are relatively homogeneous. Therefore, it is important to assess the degree to which this is the case.

One measure of effect heterogeneity across studies/estimates within a meta-analysis is tau-squared ( 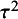 ). 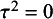 implies a single population effect, with all observed variability in estimated effects due to sampling error. Nonzero values of 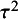 indicate heterogeneity in true effects. An advantage of 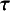 is that it is in the same units as the estimated effect, allowing one to compare the extent of heterogeneity on the same scale as the size of the effect. Table 7 indicates that only about a fourth (26%) of meta-analyses in our sample report 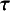 or 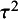 .

**Table 7:** Reporting of effect heterogeneity and median I-squared

	% reporting tau-squared	% reporting I-squared	Median I-squared
Discipline	1	2	3
Pharmacy, Therapeutics and Pharmacology	43%	96%	67%
Anatomy and Physiology	40%	88%	69%
Medicine	23%	89%	75%
Biology	20%	66%	77%
Engineering	18%	72%	78%
Public Health	26%	86%	83%
Psychology	27%	71%	84%
Education	35%	76%	86%
Business and Economics	13%	30%	89%
Environmental Sciences	15%	50%	91%

**Overall**	**26%**	**72%**	**80%**

*Note*: The data in the table are based on 100 meta-analyses for each discipline, so the percentages in the table also provide the counts (e.g., 43% = 43 meta-analyses in the given discipline). The numbers in the second column report median values across the set of meta-analyses in that discipline that report an I-squared value.

In contrast to 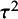 , I-squared is a relative measure of heterogeneity. It takes values between 0 and 100%^4^ and measures the percent of total variation in estimated effects that is due to true effect heterogeneity. High values of I-squared are indicative of a large degree of effect heterogeneity relative to the total variation. It is important to emphasize that I-squared is a relative measure of effect heterogeneity, not an absolute one. Two meta-analyses may exhibit the same absolute variation in true effects, but if one includes studies with less precise estimates—leading to greater total variation in observed effects—its I-squared value will be lower. Consequently, differences in I-squared can reflect not only variations in heterogeneity but also differences in sample size and study design.

Column 2 of Table 7 shows that I-squared is reported far more frequently than 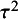 . Almost three-fourths (72%) of meta-analyses in our sample report an I-squared value, yet here again, there are differences across disciplines. For example, only 30% of meta-analyses in Business and Economics do so. However, for most disciplines, reporting I-squared is common practice. Column 3 of Table 7 displays median I-squared values, though obviously these median values only apply to the samples of studies that report I-squared. The disciplines in Table 7 are sorted from lowest median I-squared value (Pharmacy, Therapeutics and Pharmacology) to highest (Environmental Sciences).

As benchmarks for interpreting the I-squared values in Table 7, Higgins et al.’s (2003) seminal article characterizes an I-squared value of 0.75 as indicating “high” heterogeneity, while the *Cochrane Handbook for Systematic Reviews of Interventions* ^5^ classifies I-squared values above 0.75 as representing “considerable” heterogeneity. Nonetheless, Borenstein et al.^4^ caution against taking this threshold as canonical. While there are notable differences across disciplines, the common finding is that all the disciplines in our sample are characterized by substantial relative heterogeneity.

High levels of heterogeneity have several important consequences for the interpretation and reliability of meta-analytic findings. They widen prediction intervals, making average effect sizes less useful as indicators of treatment effects.^4^ They also impair the performance of meta-analytic estimators.^6^ Relatedly, tests and corrections for publication bias become less reliable in the presence of substantial effect heterogeneity.^7^ ^,^ ^8^

**Figure 6:**
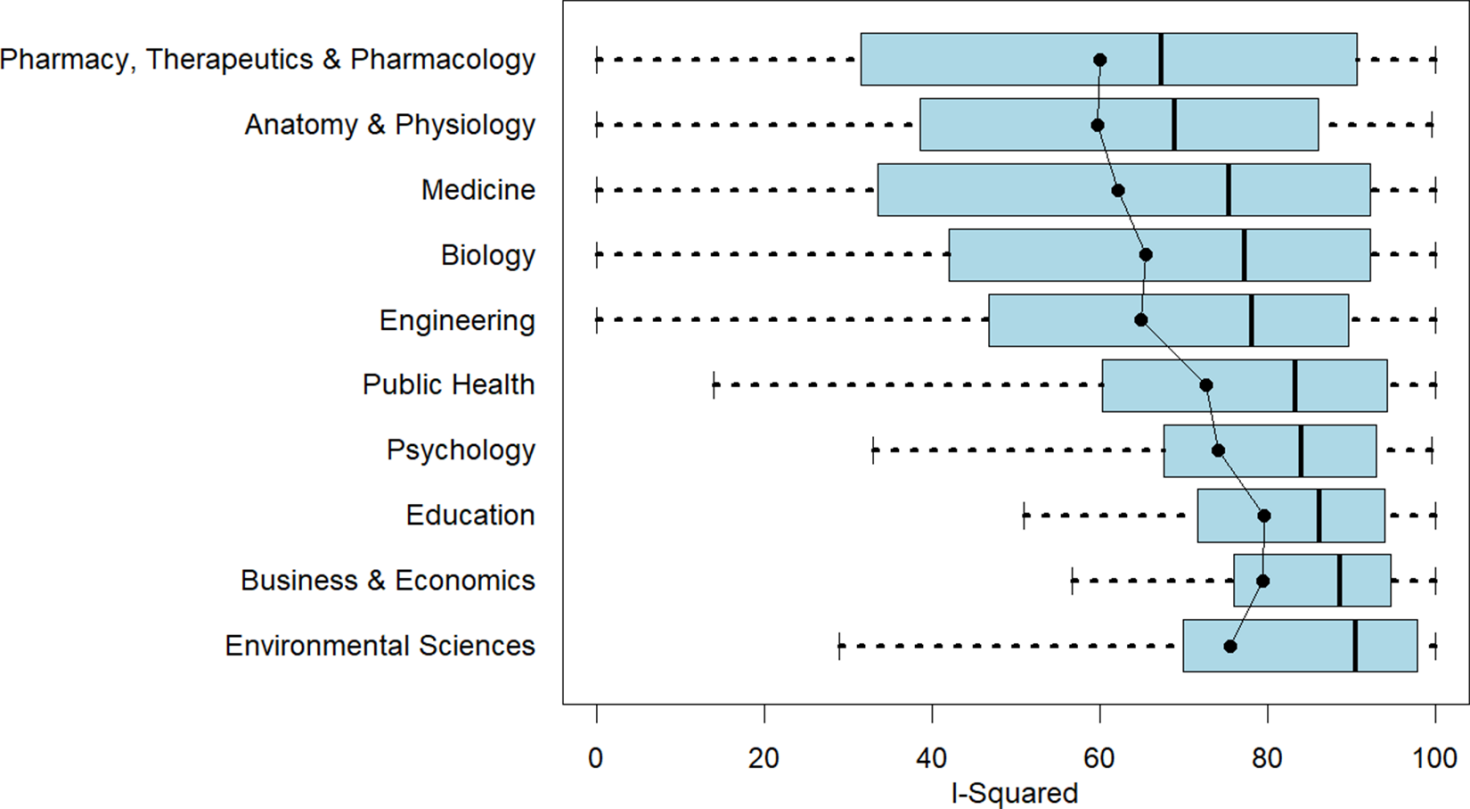
I-squared by discipline. *Note*: Figure 6 reports boxplots (without outliers) for each of the 10 disciplines with respect to I-squared as a relative measure of effect heterogeneity. Superimposed on the boxplots is the mean I-squared value for that discipline. The figure is arranged with the smallest median I-squared value at the top of the figure to the largest median I-squared value at the bottom of the figure.

Figure 6 provides a more detailed look at the distribution of I-squared within disciplines. Not only do the distributions differ in their central moments, but also in the range of their values. Meta-analyses in Pharmacy, Therapeutics and Pharmacology; Anatomy and Physiology; Medicine; and Biology run the full gamut of values with respect to relative heterogeneity. Other disciplines, especially Business and Economics, are much more concentrated towards the upper half of the scale. Because heterogeneity is a key determinant of estimator performance, capturing these differences is important for researchers designing discipline-specific Monte Carlo simulation experiments.

### 3.7 How often researchers search for publication bias—and how often they find it

One of the most significant threats to the reliability of meta-analysis is publication bias. Publication bias arises when the studies included in a meta-analysis are not representative of the broader population of research on that subject. This can occur when findings with statistically significant or positive results are more likely to be published, while studies with null or negative findings remain unpublished. As a result, the sample of included studies may be systematically skewed, leading the meta-analysis to produce a distorted and often overly optimistic summary of the empirical literature.

Researchers have developed various approaches to assess publication bias. In a later section, we report which methods for assessing publication bias are most commonly used. In this section, we merely record how often the different disciplines investigate publication bias and how often they conclude their samples have it. The corresponding results are provided in Table 8.

**Table 8:** Percent of meta-analyses testing and finding publication bias

	Testing for publication bias	Finding publication bias	No. of estimates	
			Median	Mean
Discipline	1	2	3	4
Business and Economics	74%	43%	163.5	536.7
Psychology	90%	39%	62	136.7
Anatomy and Physiology	81%	37%	19.5	31.2
Public Health	69%	35%	17.5	182.1
Biology	75%	35%	25.5	297.7
Medicine	61%	33%	17.5	26.1
Education	85%	29%	41.5	205.8
Environmental Sciences	58%	24%	63.5	184
Engineering	58%	17%	20.5	134
Pharmacy, Therapeutics and Pharmacology	74%	16%	13	23

**Overall**	**73%**	**31%**	**----**	**----**

*Note*: The numbers in Column 1 of the table are based on 100 meta-analyses for each discipline, so the percentages in the table also provide the counts (e.g., 74% = 74 meta-analyses in the given discipline). The numbers in Column 2 report conditional probabilities. For example, 43% in the first row of the table means that 43% of the 74 meta-analyses in Business and Economics (i.e., 32 of the 74 meta-analyses) that tested for publication bias concluded that there was publication bias. Columns 3 and 4 reproduce median and mean number of estimates from TABLE 1.

Column 1 of Table 8 reports the percent of meta-analyses in each discipline that test for publication bias, where “test” includes qualitative tests such as the funnel plot. Overall, approximately three-fourths (73%) of the meta-analyses assess publication bias. Column 2 shows how often they conclude that publication bias is present. The disciplines in the table are ordered from those where evidence for publication bias appears to be most prevalent (Business and Economics) to those where it is found least often (Pharmacy, Therapeutics and Pharmacology).

Given the widespread concern with publication bias, it is notable that some disciplines do not routinely evaluate it. While 9 out of 10 meta-analyses in Psychology investigate publication bias, the rates in Medicine, Environmental Sciences, and Engineering are closer to 6 out of 10. One might think the latter result is partly a function of the number of studies/estimates per meta-analysis, but Anatomy and Physiology has relatively few studies/estimates per meta-analysis (see Table 1), and yet 81% of the meta-analyses in that discipline assess publication bias.

It is notable that most assessments for publication bias do not conclude that it is present. Of the 725 meta-analyses that evaluated publication bias, only 31% found evidence of it. In no discipline was the null hypothesis of no publication bias rejected more than 50% of the time. In Engineering and in Pharmacy, Therapeutics and Pharmacology, rejection rates were below 20%. We note that experimental and observational studies show little difference in either the frequency of testing for publication bias or the likelihood of detecting it (cf. Appendix A).

Of course, the moderately low probabilities of detecting publication bias should be interpreted with caution. It is well known that many tests for publication bias suffer from low power, especially when sample sizes are small.^9^ ^–^ ^11^ Thus, failure to find evidence of publication bias does not necessarily mean that publication bias is absent.

To investigate this further, Columns 3 and 4 reproduce the median and mean numbers of estimates from Table 1. There is some evidence that the probability of finding evidence for publication bias is increasing in sample size. We then examined this more formally by regressing the likelihood of detecting publication bias on the number of estimates for the 731 meta-analyses that conducted such tests. Although the relationship was positive, the estimated effect was small. The estimate from a univariate, linear probability model indicated that an increase in sample size of 100 estimates was associated with a 0.5 percentage point increase in the probability of detecting publication bias. Adding discipline-specific fixed effects and estimating corresponding probit models produced estimates of similar magnitude.

Ultimately, we cannot determine whether the relatively low probabilities of detecting publication bias reflect limited statistical power or whether publication bias is genuinely less prevalent than commonly asserted.^12^ ^,^ ^13^

### 3.8 Methods used to detect publication bias

In this section, we report the methods that researchers employ to detect publication bias. We categorize these as follows:

- Funnel plots^14^
- Egger’s regression, including variants of FAT-PET PEESE^9^ ^,^ ^15^
- Trim and Fill^16^
- Begg and Mazumdar’s rank correlation test^17^
- Fail Safe N test^18^
- Selection model tests^19^
- p-uniform and p-curve tests^20^ ^,^ ^21^
- Other

**Figure 7:**
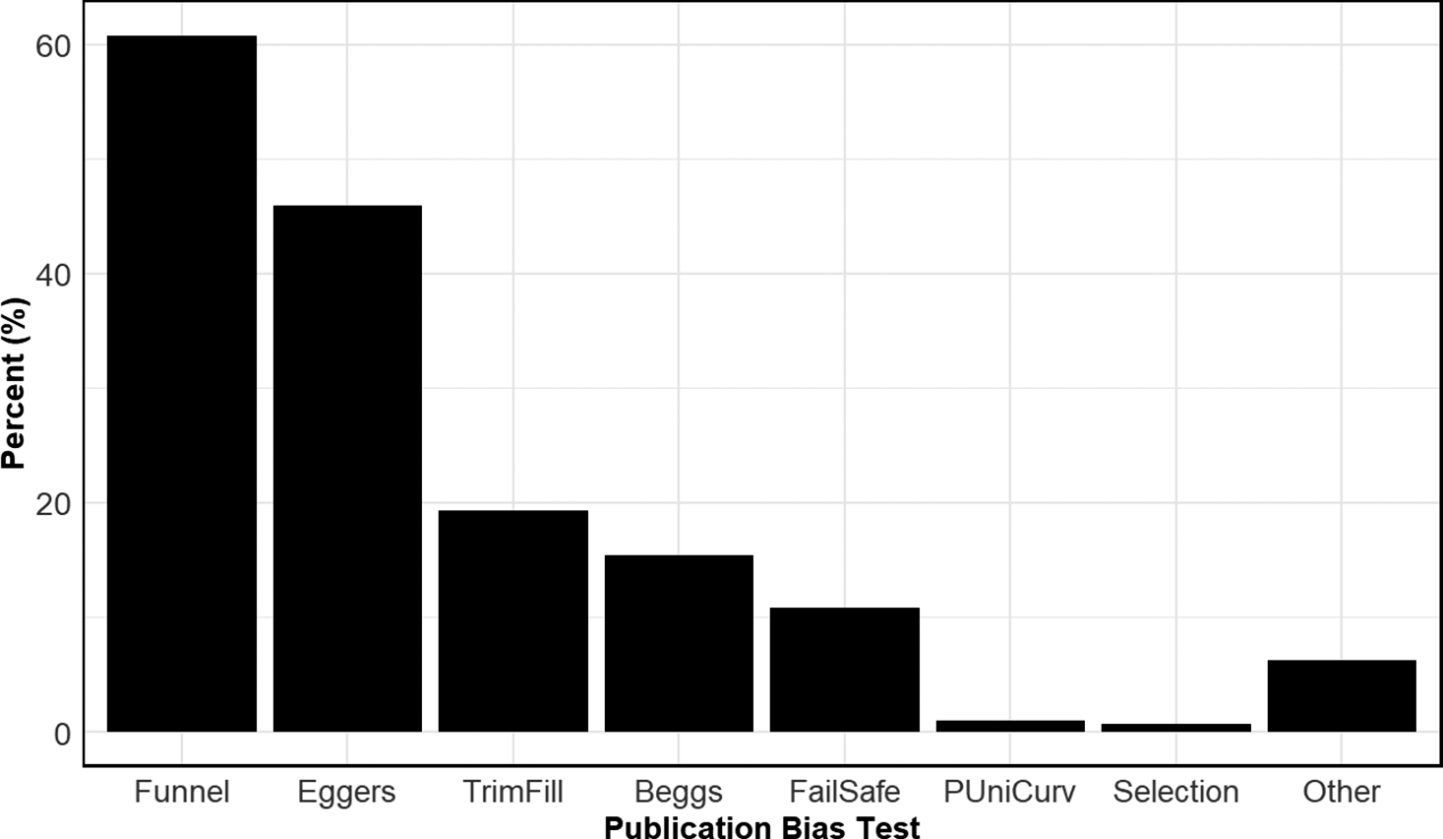
Prevalence of different types of tests for publication bias. *Note*: Figure 7 reports aggregate usage rates of different types of publication bias tests. Funnel, Funnel plot; Eggers, Egger-type regression; TrimFill, Trim and Fill; Beggs, Begg and Mazumdar’s rank correlation test; FailSafe, Fail Safe N; Selection, publication bias uses a selection model; PUniCurv, either p-Uniform or p-Curve test for publication bias.

Figure 7 shows the prevalence of different methods used to detect publication bias across disciplines. By far the most commonly used approach is the funnel plot, employed in over 60% of all meta-analyses in our sample. The second most frequent method is Egger’s regression test and its variants, used in approximately 46% of cases. Beyond these, the use of alternative methods drops off substantially.

Among the various approaches for assessing publication bias, the funnel plot is distinctive in being a purely qualitative method. For this reason, Table 9 separates meta-analyses, by discipline, into three categories: those that rely solely on funnel plots, those that rely solely on other quantitative methods, and those that use both (Columns 1–3, respectively). Each cell in these columns reports two numbers. The top number indicates the unconditional probability that a meta-analysis from a given discipline uses the corresponding category of publication bias assessment. For example, 11% of all meta-analyses in Anatomy and Physiology rely solely on funnel plots. Ten percent rely solely on alternative quantitative methods, while 59% combine one or more quantitative tests with a funnel plot.

**Table 9:** Assessing publication bias: Funnel plots and other approaches

	Only funnel	Other than funnel	Funnel + other
Discipline	1	2	3
Anatomy and Physiology	11% *(14%)*	10% *(13%)*	59% *(74%)*
Biology	12% *(16%)*	9% *(12%)*	55% *(72%)*
Business and Economics	4% *(5%)*	25% *(34%)*	45% *(61%)*
Education	5% *(6%)*	14% *(17%)*	64% *(77%)*
Engineering	14% *(24%)*	5% *(9%)*	39% *(67%)*
Environmental Sciences	10% *(17%)*	15% *(25%)*	34% *(58%)*
Medicine	15% *(23%)*	11% *(17%)*	38% *(59%)*
Pharmacy, Therapeutics and Pharmacology	26% *(36%)*	4% *(5%)*	43% *(59%)*
Psychology	5% *(6%)*	16% *(18%)*	69% *(77%)*
Public Health	14% *(20%)*	9% *(13%)*	46% *(67%)*

**Overall**	**12%** ***(16%)***	**12%** ***(16%)***	**49%** ***(68%)***

*Note*: The top numbers in the first three columns of the table are based on 100 meta-analyses for each discipline, so the percentages in the table also provide the counts. Thus, 11% = 11 meta-analyses in Anatomy and Physiology use only a funnel plot to assess publication bias; 10% = 10 meta-analyses use something besides a funnel plot; and 59% = 59 meta-analyses use a combination of both. Thus, 80 meta-analyses in Anatomy and Physiology assess publication bias in some way. The italicized number below the top number is the conditional probability. Conditional on assessing publication bias, it shows what percent of meta-analyses use each of the three approaches. Thus, among all meta-analyses in Anatomy and Physiology that assess publication bias, 14% solely use funnel plots, 13% only use quantitative tests other than funnel plots, and 74% use a combination of both. The last column reports the discipline mean number of tests employed by meta-analyses that assess publication bias. This number includes funnel plots. For example, meta-analyses in Anatomy and Physiology that test for publication bias use an average of 2.4 tests.

The italicized number below the top number represents the conditional probability—that is, conditional on testing for publication bias, it indicates the percentage of meta-analyses that use each of the three approaches. For example, among the 81 meta-analyses in Anatomy and Physiology that assess publication bias, 14% rely solely on funnel plots, 13% use only quantitative methods other than funnel plots, and 74% combine both approaches.

The table shows that 68% of meta-analyses that assess publication bias use a combination of funnel plots and quantitative tests. A few disciplines stand out for their exclusive reliance on funnel plots. In Pharmacy, Therapeutics and Pharmacology, over a third (36%) of meta-analyses that investigate publication bias rely solely on a funnel plot. Similarly, approximately a quarter of meta-analyses in Engineering and Medicine also only use funnel plots to determine whether their samples exhibit publication bias.

Table 10 explores in greater detail the different methods used by meta-analyses to detect publication bias. It breaks down the aggregate prevalence rates from Figure 7 into discipline-specific numbers. The discipline numbers mostly follow the aggregate rates. Funnel plots and Egger regression tests dominate the other types of assessment methods.

**Table 10:** Prevalence of methods to assess publication bias

	Funnel	Eggers	TrimFill	Beggs	FailSafe	PUniCurv	Selection	Other	No. of methods
Discipline	1	2	3	4	5	6	7	8	9
Anatomy and Physiology	70%	56%	28%	26%	8%	0%	0%	2%	1.4
Biology	67%	51%	15%	17%	8%	1%	0%	4%	1.3
Business and Economics	49%	42%	15%	7%	22%	3%	5%	20%	1.7
Education	69%	52%	29%	17%	20%	2%	0%	15%	1.7
Engineering	53%	36%	13%	13%	5%	0%	0%	0%	1.3
Environmental Sciences	44%	36%	11%	13%	17%	0%	0%	5%	1.4
Medicine	53%	36%	15%	13%	2%	0%	0%	2%	1.3
Pharmacy, Therapeutics and Pharmacology	69%	45%	4%	20%	1%	0%	0%	0%	1.1
Psychology	74%	58%	45%	8%	21%	4%	2%	13%	1.8
Public Health	60%	47%	18%	20%	4%	0%	0%	1%	1.3

**Overall**	**61%**	**46%**	**19%**	**15%**	**11%**	**1%**	**1%**	**6%**	**1.5**

*Note*: Columns 1–8 in the table are based on 100 meta-analyses for each discipline, so the percentages also provide the counts (e.g., 70% = 70 meta-analyses in the given discipline). Note that the sum of the percentages (counts) across each row are greater than 100% (100 meta-analyses) because studies can use more than one method to detect publication bias. Funnel, Funnel plot; Eggers, Egger-type regression; TrimFill, Trim and Fill; Beggs, Begg and Mazumdar’s rank correlation test; FailSafe, Fail Safe N; Selection, publication bias uses a selection model; PUniCurv, either p-Uniform or p-Curve test for publication bias. Column 9 reports the average number of different methods for testing publication bias, where Funnel, Eggers, and Beggs are counted as one method since they all use the relationship between the effect size and its standard error as a measure of publication bias.

A few disciplines are noteworthy for their disproportionate use of particular methods. For example, almost half of all meta-analyses in Psychology (45%) use Trim and Fill. Fail Safe N is mostly used in Business and Economics, Education, Environmental Sciences, and Psychology. And Business and Economics, Education, and Psychology are noteworthy for using a wider variety of methods not employed by other disciplines (e.g., testing for differences between published and unpublished studies; using year of publication to test for time-lag bias).^22^ The last column reports the average number of methods to test for publication bias, where we count Funnel. Eggers and Beggs as the same method since they all use the relationship between the effect size and its standard error as a measure of publication bias.

On average, meta-analyses employ 1.5 distinct methods to test for publication bias. However, the distribution of methods used is positively skewed, with only about one-third of studies applying two or more distinct approaches.

### 3.9 Types of statistical software packages

One other dimension on which meta-analyses differ is the statistical software packages they use. We tracked usage of the following packages:

- R, especially the packages “meta,” “metafor,” “robumeta,” and “dmetar”: free, open-source software environment produced by the R Foundation for Statistical Computing
- Stata, especially the “meta” suite of commands: proprietary software produced by StataCorp
- RevMan (“Review Manager”): free software developed by the Cochrane Collaboration
- CMA (“Comprehensive Meta-Analysis”): proprietary software produced by Biostat
- SPSS: proprietary software produced by IBM
- JASP: free, open-source software produced by researchers at the University of Amsterdam
- Other

**Figure 8:**
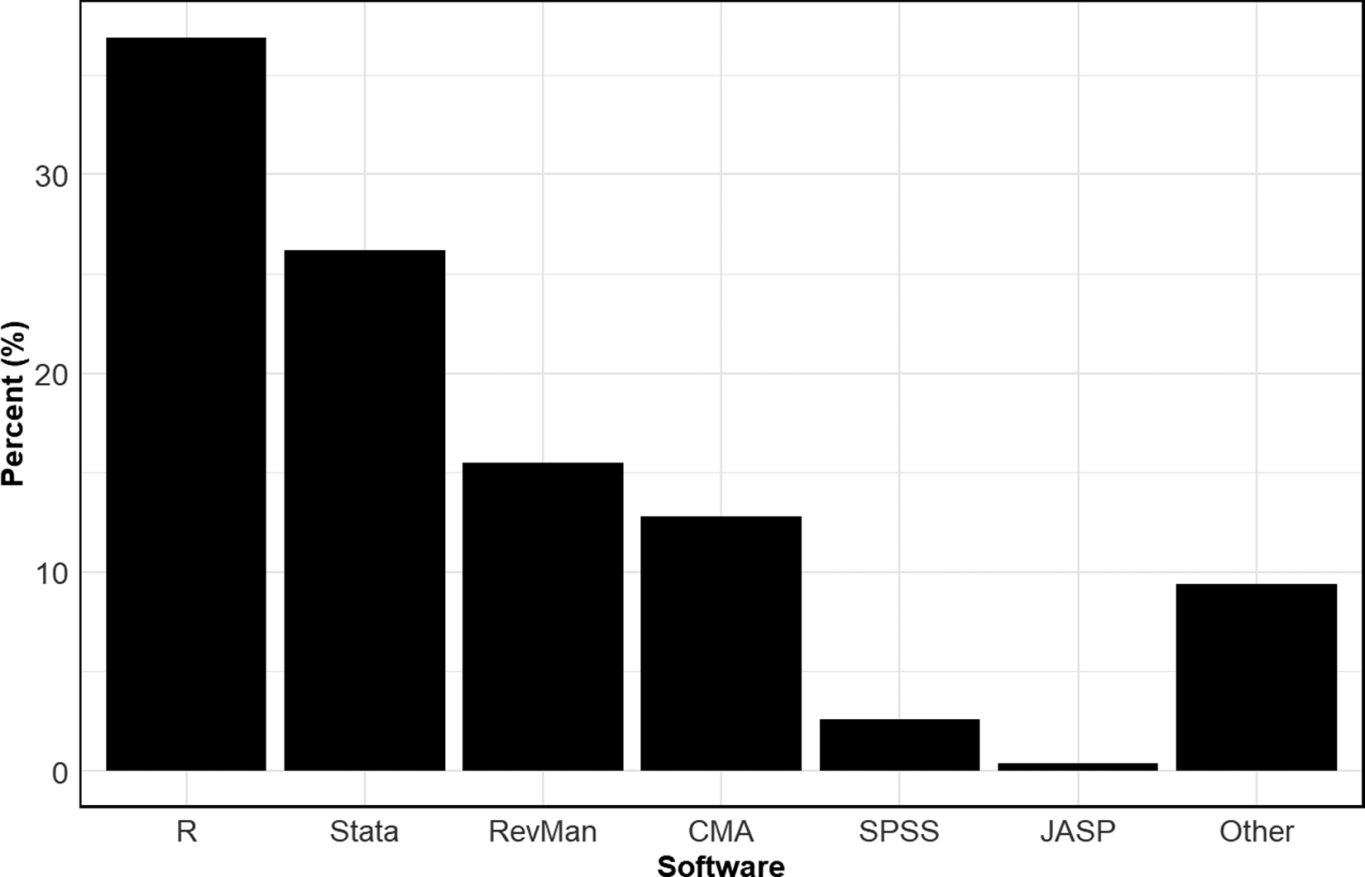
Usage rates of different statistical packages. *Note*: Figure 8 reports aggregate usage rates of different statistical software packages.

Figure 8 reports the aggregate usage rate of the different statistical software packages across all disciplines. A wide variety of packages are used. The most common is R, used by over a third of all meta-analyses. The next most common is Stata, used by approximately a quarter of the meta-analyses in our sample. The next most common, in order, are RevMan, CMA, SPSS, and JASP.

Table 11 breaks down the overall usage rates by discipline. There are clear differences across the disciplines. While R is the most used statistical package for most disciplines, Stata is preferred in Medicine and Public Health, and RevMan is the most employed package in Pharmacy, Therapeutics, and Pharmacology. R, Stata, and RevMan are approximately evenly used in Astronomy and Physiology and Engineering. We note that researchers wishing to have their systematic reviews included in the Cochrane Database of Systematic Reviews are strongly encouraged to use RevMan.

**Table 11:** Prevalence of different statistical packages

Discipline	R	Stata	RevMan	CMA	SPSS	JASP	Other
Anatomy and Physiology	28%	26%	28%	16%	0%	1%	11%
Biology	55%	28%	15%	6%	3%	0%	8%
Business and Economics	33%	12%	2%	9%	2%	0%	12%
Education	38%	19%	11%	26%	1%	1%	6%
Engineering	26%	28%	26%	10%	5%	0%	14%
Environmental Sciences	48%	27%	4%	4%	4%	0%	13%
Medicine	29%	38%	12%	17%	2%	0%	11%
Pharmacy, Therapeutics and Pharmacology	26%	36%	42%	6%	2%	1%	8%
Psychology	54%	5%	1%	29%	5%	1%	4%
Public Health	32%	43%	14%	5%	2%	0%	7%

**Overall**	**37%**	**26%**	**16%**	**13%**	**3%**	**0%**	**9%**

*Note*: The data in the table are based on 100 meta-analyses for each discipline, so the percentages in the table also provide the counts (e.g., 28% = 28 meta-analyses in the given discipline). Note that the sum of the percentages (counts) across each row can be greater than 100% (100 meta-analyses) because studies sometimes use more than one statistical package.

The choice of statistical package is consequential. For example, the use of point-and-click packages such as CMA, RevMan, and JASP can be restrictive because users are limited by the built-in functionality of those packages. Furthermore, proprietary packages such as CMA and Stata may be problematic for environments that encourage the sharing of data and code, as researchers interested in reproducing results from these meta-analyses may not have access to those packages.

## 4 Improving core practices in meta-analysis: Evidence and recommendations

In this section, we draw observations from the preceding sections and offer a set of recommendations to improve meta-analytic practice. These recommendations are not intended to be exhaustive. Numerous existing guidelines and best practice articles already support researchers conducting meta-analyses.^23^ ^–^ ^26^ Many are tailored to specific disciplines, such as psychology,^27^ ^–^ ^29^ health,^30^ ^,^ ^31^ economics,^32^ ^,^ ^33^ education,^34^ ^,^ ^35^ and medicine.^36^ ^,^ ^37^

We note that several of our recommendations echo points already raised in earlier work. Still, they warrant restating, as it is clear that in several important respects, current practice falls short of what established guidelines and best practices recommend. That said, we acknowledge that meta-analyses differ in their goals, and while our recommendations are broadly applicable, they may not be suitable for every case.

### 4.1 Underutilization of meta-regression to investigate heterogeneity

Numerous guidelines and best practice articles recommend the use of meta-regression to investigate effect heterogeneity.^33^ ^,^ ^34^ ^,^ ^38^ Table 7 shows that all disciplines in our sample exhibit “high” median heterogeneity according to Higgins et al.,^39^ and “considerable” median heterogeneity based on the criteria of Deeks et al.^5^ While both subsample analysis and meta-regression are valuable tools for investigating heterogeneity, we focus on meta-regression because—unlike subsample analysis—it allows for the estimation of the unique contribution of each characteristic while controlling for the influence of others.

In meta-analyses, a common goal is to estimate the overall mean of the effect of a treatment or intervention. Meta-regression expands this analysis by adding explanatory variables to the equation. It enables an analysis of how factors related to study design, data characteristics, and estimation methods influence the magnitude of estimated effects, thereby identifying which characteristics contribute most to heterogeneity among these estimates.

Column 1 of Table 12 reports the percent of meta-analyses that estimate meta-regressions for each discipline in our sample. In this analysis, to be counted as a meta-regression, the estimated effect size needed to be regressed on some sample, study, or estimation characteristics other than the standard error variable. We did not count univariate, Egger-type regressions as “meta-regressions” because we wanted to focus on the use of meta-regression as a tool for explaining heterogeneity in effect sizes, rather than as a tool to test for publication bias.

**Table 12:** Use of meta-regression in meta-analyses

	% estimating meta-regressions	% MRs with > 1 regressor	Median I-squared	Median no. of studies	Median no. of estimates
Discipline	1	2	3	4	5
Medicine	21%	29%	75%	16	17.5
Pharmacy, Therapeutics and Pharmacology	31%	32%	67%	11	13
Public Health	31%	29%	83%	15	17.5
Engineering	35%	31%	78%	15.5	20.5
Environmental Sciences	42%	24%	91%	22.5	63.5
Anatomy and Physiology	47%	21%	69%	14	19.5
Biology	49%	27%	77%	21	25.5
Business and Economics	76%	79%	89%	53.5	163.5
Psychology	77%	14%	84%	32	62
Education	81%	40%	86%	28.5	41.5

**Overall**	**49%**	**35%**	**80%**	**21**	**28**

*Note*: The data in the first column of the table are based on 100 meta-analyses for each discipline, so the percentages also provide the counts (e.g., 21% = 21 meta-analyses in the given discipline). The values in the Column 2 report the percentage of meta-regressions that have more than one regressor. The median I-squared values in Column 3 are reproduced from, and explained in, Table 7. The median numbers of studies and estimates in Columns 4 and 5 are reproduced from, and explained in, Table 1.

The disciplines in Table 12 are ordered from the least frequent use of meta-regression (Medicine) to the most frequent use (Education). The prevalence of meta-regression varies widely across disciplines. Outside the disciplines of Business and Economics, Psychology, and Education, half or less of all meta-analyses make use of meta-regression to investigate the sources of heterogeneity in estimated effects.

Of these, most are univariate regressions where the estimated effect is regressed on a single sample, study, or estimation characteristic (cf. Column 2). For example, only 21% of meta-analyses in Medicine estimated a meta-regression. Of these, only 29% used more than one variable to explain the heterogeneity in estimated effects; that is, a total of six meta-analyses (= 21 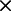 0.29). In fact, other than Business and Economics, univariate meta-regressions comprised the overwhelming majority of meta-regressions across disciplines.

One possible reason for this is that different disciplines may face different benefits and costs in conducting meta-regressions. Column 3 of Table 12 reproduces the median I-squared values from Table 7. One might think that the disciplines with the most heterogeneity would also have the greatest incentive to conduct meta-analyses. However, the use of meta-regression does not appear to be related to the discipline levels of heterogeneity.

Columns 4 and 5 of Table 12 report the median number of primary studies and estimates by discipline (reproduced from Table 1). A clear positive relationship emerges between the use of meta-regression and the size of the meta-analytic sample. This is consistent with the explanation that the limited use of meta-regression in some disciplines is due to their meta-analyses including too few estimates. Appendix B investigates this possibility and finds that even in disciplines with relatively small-sized meta-analyses, there is scope for greater use of meta-regression.

An additional advantage of estimating meta-regressions is their ability to predict effect sizes conditional on specific treatment and outcome characteristics. This allows researchers to estimate how effective a treatment would be under “preferred” or “best-practice” conditions. Meta-regression can also be used in conjunction with risk of bias tools such as RoB 2^40^ and ROBINS-I,^41^ which assesses the quality and credibility of primary studies. When risk of bias is coded as a study-level moderator and incorporated into the meta-regression, researchers can explore whether and how effect sizes vary systematically with study quality. This can support the identification of treatment effects that are less likely to be inflated by methodological shortcomings—providing a more credible basis for estimating what might happen under optimal conditions.

Despite its potential, the calculation of such “preferred” or “best-practice” estimates from meta-regressions remains significantly underutilized. Among the 1,000 meta-analyses in our sample, only eight reported estimates explicitly derived under best-practice assumptions—seven in Business and Economics and one in Psychology.

In summary, despite substantial heterogeneity being a pervasive characteristic of meta-analyses, the use of meta-regression is relatively limited. In only three disciplines—Business and Economics, Psychology, and Education—did more than 50% of meta-analyses use this tool. Furthermore, the great majority of meta-regressions consist of univariate regressions where the estimated effect sizes are regressed on a single sample, study, or estimation characteristic. This leads to the following recommendations.

**Recommendation #1a:** *Meta-analysts should increase their use of meta-regression to explore sources of effect heterogeneity and develop “best-practice” estimates whenever sample size and data conditions allow.*

**Recommendation #1b:** *Analysts should go beyond the use of single variable meta-regression and include multiple covariates where possible.*

### 4.2 The use of weights that are dependent on effect size estimates

A central assumption of meta-analytic models is that the weights and effect size estimates are independent. However, in some cases, the variance (i.e., the inverse of the weights) is a function of the effect size parameter, creating dependence between them. This functional dependence occurs for several types of effect sizes, such as Cohen’s d, but is generally considered most problematic for correlations.^33^ ^,^ ^42^ This is evident from the standard error formulas below. The standard error of *r* is given by^43^
(1.a)

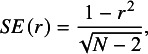

 and the standard error for *PCC* is similar^44^:
(1.b)

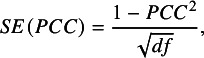

 where *df* equals the degrees of freedom from the respective regression equation. These formulas make explicit that the correlation standard error is mathematically dependent on the effect size estimate.

At least two problems stem from the fact that the standard error is a mathematical function of the estimated effect size. The first concerns the calculation of the overall mean effect. Meta-analyses that employ inverse-variance weighting use the standard error to determine how much weight each estimate receives in the computation of the mean effect. As shown in Equations (1.a) and (1.b), larger estimates yield smaller standard errors. Consequently, larger estimates receive greater weight in the meta-analysis, which leads to upwardly biased estimates of the overall mean.^45^ ^,^ ^46^

A second problem arises when tests for publication bias depend on the standard error, as in Egger-type regressions, Begg and Mazumdar’s rank correlation tests, and funnel plots. Equations (1.a) and (1.b) highlight that these tests may detect a spurious relationship between the estimated correlation and its standard error even when no publication bias is actually present. This can lead to incorrect inferences about the existence of publication bias. One solution to this problem is to transform correlations to Fisher’s *z* values.^47^ Another is to weight observations using a sample size-based weight that is independent of the observation’s correlation value.^23^ We denote this approach as “N-weights.”

As previously shown (cf. Figure 4), correlations, including Pearson’s product–moment correlation (*r*) and partial correlation (*PCC*), are used in approximately 10% of the meta-analyses in our sample. Columns 1–3 of Table 13 focus on estimates of the overall mean effect size. Column 1 reports how often a meta-analysis uses a correlation for an effect size. Column 2 shows that Fisher’s z estimates are rarely employed for robustness checking. Only 4% of the meta-analyses that use correlations for estimation also report Fisher’s *z* estimates.

**Table 13:** Correlations, Fisher’s z, and alternative weights

	Effect sizes		Weights	Testing for publication BIAS		
	Correlation and Fisher’s *z*			Egger’s (E), Begg’s (B), Funnel plot (F)		
	Count	Fisher’s *z*	N-weights	Count	E/B/F	E/B
Discipline	1	2	3	4	5	6
Anatomy and Physiology	2	0%	0%	0	–	–
Biology	1	0%	100%	1	0%	0%
Business and Economics	46	9%	50%	36	81%	58%
Education	13	0%	23%	10	70%	60%
Engineering	6	0%	17%	2	100%	0%
Environmental Sciences	5	0%	0%	3	33%	33%
Medicine	0	–	–	0	–	–
Pharmacy, Therapeutics and Pharmacology	0	–	–	0	–	–
Psychology	23	0%	48%	21	62%	38%
Public Health	3	0%	33%	3	100%	67%

**Overall**	**99**	**4%**	**40%**	**76**	**72%**	**50%**

*Note*: Columns 1 reports how many meta-analyses in the respective disciplines have an effect size that is a correlation. Column 2 reports how often the respective meta-analyses also use a Fisher’s z effect size as a robustness check. Column 3 reports how often meta-analyses that use correlations use sample size-based weights that are independent of observation-level variation in correlations. Column 4 reports how many meta-analyses have an effect size that is a correlation and also test for publication bias. Column 5 reports how often these meta-analyses used an Egger’s type test (E), a Begg’s rank correlation test (B), or a funnel plot (F) to test for publication bias. Column 6 does the same as Column 5 but only reports if the meta-analyses used an Egger’s type test (E) or Begg’s rank correlation test (B).

An alternative to Fisher’s *z* is to use N-weights. Column 3 reports that this is a more common strategy than transforming correlations into Fisher’s *z*. In Business and Economics and Psychology, where the use of correlations is most prevalent, approximately half of all meta-analyses use “N-weights” rather than inverse variance weights. Overall, 56% of meta-analyses that use correlations as the main effect size do not use N-weights or attempt a robustness check by transforming correlations to Fisher’s *z*. This leads to the following recommendation.

**Recommendation #2a:** *Meta-analyses need to ensure that weights are not a function of the effect size estimates (e.g., the correlation coefficient r). To mitigate this, either a variance-stabilizing transformation should be used (e.g., Fisher’s z^47^ ^,^ ^48^) that ensures the variance (and thus weights) are independent of the effect size estimates, or alternative weights should be chosen that are not a function of the effect size (e.g., N-weights^23^).*

The last three columns of Table 13 highlight concerns with using the standard error of the correlation in Egger regressions, Begg and Mazumdar’s rank correlations, and funnel plots. In these cases, the functional dependence between correlations and their standard errors can lead to misleading evidence of publication bias, even when no such bias exists.

Column 4 reports the number of meta-analyses that use correlation-based effect sizes and also conduct a publication bias test. Column 5 shows the percentage of those studies that rely on standard error–based methods—namely, Egger’s test, Begg’s test, or funnel plots. Column 6 narrows the focus further by reporting the percentage that use formal quantitative tests, specifically Egger’s or Begg’s.

For example, in Business and Economics, 36 meta-analyses used correlations as effect sizes and also tested for publication bias. Of these, 81% relied on standard error–based methods, where the standard error was functionally related to the magnitude of the correlation. 58% of the formal tests (Egger’s or Begg’s, excluding funnel plots) used the correlation standard error to indicate the presence of publication bias.

While these patterns vary across disciplines, the overall conclusion is clear: meta-analyses that use correlations as effect sizes frequently rely on publication bias tests that depend on the standard error of the correlation—even though the standard error is functionally linked to the effect size itself, regardless of whether publication bias is present. This motivates the following recommendation:

**Recommendation #2b:** *Researchers using correlations as effect sizes and testing for publication bias should avoid relying on the correlation’s standard error. Instead, they should either (i) transform correlations to Fisher’s z scale and use its corresponding standard error, (ii) replace the standard error with a measure of sample size,^49^ or (iii) apply instrumental variable estimation with sample size as an instrument for the endogenous standard error.^50^*

### 4.3 Limited correction for publication bias

Best practice guidelines recommend that researchers adjust their estimates when publication bias is present.^29^ ^,^ ^50^ ^,^ ^51^ In this section, we assess the extent to which meta-analyses follow this guidance. Columns 1 and 2 of Table 14 reproduce the discipline-level results from Table 8, showing how often meta-analyses tested for and found evidence of publication bias. Column 3 indicates whether those meta-analyses subsequently attempted to adjust their estimates after detecting bias.

**Table 14:** Correcting for publication bias

	Percent testing	Percent finding	Percent correcting(1)	Percent correcting(0)
Discipline	1	2	3	4
Anatomy and Physiology	81%	37%	40%	19%
Biology	75%	35%	42%	8%
Business and Economics	74%	43%	75%	18%
Education	85%	29%	52%	27%
Engineering	58%	17%	50%	9%
Environmental Sciences	58%	24%	29%	6%
Medicine	61%	33%	35%	11%
Pharmacy, Therapeutics and Pharmacology	74%	16%	0%	6%
Psychology	90%	39%	63%	37%
Public Health	69%	35%	42%	12%

**Overall**	**72%**	**31%**	**47%**	**14%**

*Note*: Columns 1 and 2 of the table reproduce Columns 1 and 2 from Table 8. Column 3 reports the percent of meta-analyses finding publication bias that then went on to correct for it. Column 4 reports the percent of meta-analyses that did not find publication bias that still went on to correct for it.

In most disciplines, the majority of meta-analyses that identified publication bias did not proceed to adjust their estimates. For example, in Anatomy and Physiology, 81% of meta-analyses tested for publication bias, and 37% of those detected it. However, only 40% of the meta-analyses that found publication bias subsequently corrected their original estimates (“Percent Correcting(1)”). Only Business and Economics (75%) and Psychology (63%) adjusted for publication bias in more than half of the cases where it was detected.

Overall, less than half (47%) of meta-analyses that found evidence of publication bias adjusted their estimates of the overall mean effect (see Column 3). Among meta-analyses that did not reject the null hypothesis of no publication bias, only 14% made any correction—effectively accepting the null hypothesis without further adjustment.

**Recommendation #3a:** *Meta-analyses should correct for publication bias when tests indicate its presence. As a robustness check, they should also correct for publication bias even when they fail to confirm its presence because power is typically low^52^ and tests may not signal it even when it is there. Bias-corrected estimates should be prominently highlighted when reporting results.*

When applying regression-based methods to correct estimates of the overall mean effect—such as the PET-PEESE approach advocated by Stanley and Doucouliagos^53^—the meta-analyses in our sample relied almost exclusively on univariate regression models, using the standard error as the sole explanatory variable. Potentially relevant sample, study, or estimation characteristics that might be correlated with the standard error were rarely included. Incorporating these characteristics in multivariate regression models may better isolate the influence of publication selection, leading to more accurate and reliable corrections of the overall mean effect.^54^.

Another important factor in both assessing and correcting publication bias is heterogeneity. As noted earlier, high levels of effect heterogeneity are pervasive across all disciplines in our sample (Table 7). Under such conditions, regression-based corrections for publication bias become less reliable.^8^ There is evidence that selection models may outperform regression methods in these settings.^6^ ^,^ ^7^ Specifically, in the presence of heterogeneity, methods other than selection models can signal publication bias even when no such bias exists. Despite this, selection models remain relatively underused (cf. Table 10). In light of the above, we offer the following recommendations.

**Recommendation #3b:** *Given concerns about omitted variable bias and high heterogeneity, selection models and multivariate regression models should be considered as robustness checks when correcting publication selection.*

Research has shown that no single method is universally optimal for correcting publication bias.^6^ ^,^ ^55^ Thus, it is important that researchers employ a variety of approaches when testing and correcting for it. Column 9 of Table 10 reports the average number of different approaches used by each discipline to test for publication bias. Funnel plots, Egger’s test, and Begg’s test are counted as variants of the same approach, since all three rely on the relationship between the effect size and its standard error to detect publication bias.

On average, disciplines employed 1.5 methods for testing publication bias, with no discipline exceeding an average of two. However, the average gives a misleading impression of how common multiple testing actually is, as the right-skewed distribution is driven by a minority of meta-analyses using several methods. Among all meta-analyses that tested for publication bias, just over one-third (37%) employed two or more methods. Of the 10 disciplines, only Education and Psychology used multiple methods in more than half of their meta-analyses (59% and 62%, respectively). The next recommendation follows accordingly.

**Recommendation #3c:** *Recognizing that no single method for correcting publication bias is universally optimal,^6^ ^,^ ^55^ researchers should draw on recent research and select two or more different approaches. Given the current lack of consensus in this area, we call for more research that can guide meta-analysts in selecting the most appropriate methods for addressing publication bias.*

### 4.4 Insufficient accommodation for dependency among estimates

Best-practice guidelines recommend that meta-analyses address statistical dependency among effect sizes, particularly when multiple estimates are derived from a single primary study.^30^ ^,^ ^33^ ^,^ ^42^ As shown in Table 2, many meta-analyses include more than one estimate per study. There are two general types of dependence: (1) measured on the same people (“correlated effects”) and (2) nested in the same study. The first has to do with estimation error while the second has to do with random effects. When dependence is not accounted for properly in a meta-analysis—for example, by using methods that require independence—estimates are inefficient and the Type I error of the hypothesis tests is incorrect. In general, this results in tests that reject null hypotheses more often than they should.

With respect to estimator efficiency, there are various approaches to addressing this dependence, depending upon its type. When effect sizes are measured on different people but are nested in the same study, then a Multilevel Model (MLM) is an appropriate approach. The most common of which is three-level meta-analysis.^56^ A difficulty with this approach is that it requires a large number of studies.

Importantly, the MLM approach is only valid when there is no dependence induced from estimation, that is, from effect sizes measured on the same individuals (cf. (1) above). When this type of dependence arises, a different approach is needed. One approach is multivariate meta-analysis (MVMA^57^). In this approach, the dependence structure is modelled directly in terms of a variance–covariance matrix. While this approach has been available for a long time, a difficulty with implementation is that it requires estimates of the correlation between effect sizes. These correlations, however, are often not provided in primary studies. When this is the case, this model-based approach can be incorrect, again leading to inflated Type I errors.

An alternative approach to dependency is to prioritize the accuracy of standard error estimation over the efficiency of coefficient estimates and use clustered standard errors. Cluster-Robust Standard Errors (CRSE) can be applied in any regression framework. In the context of meta-analysis, Robust Variance Estimation (RVE) offers a specialized form of CRSE that accounts for the weighting schemes and sample size structures typical of meta-analytic data.^58^ ^,^ ^59^ The theoretical foundation for both CRSE and RVE is asymptotic, where it can be demonstrated that estimators converge to the correct standard errors as the number of studies becomes large.

Various adjustments have been developed to improve the performance of CRSE estimators when there are small to moderate numbers of studies. The oldest adjustment—called CR1—involves a simple multiplicative adjustment to the standard errors (e.g., *m/*(*m-p*)); this is the default correction included in regression package implementations of CRSE in software, including Stata. An alternative correction—called CR2—is known as the bias-adjusted, cluster–robust variance estimator or small-sample corrected cluster-robust variance estimator.^59^ ^–^ ^62^ This CR2 correction is the default in meta-analysis software with RVE implementations in both R and Stata.

The CR2 adjustment has been shown to have better inference properties when the number of clusters (e.g., studies) is small, while the CR1 estimator has been shown to have inflated Type I errors, even with as many as 50 or 70 studies. Since the CR2 adjustment also performs well when the number of clusters is large, it should generally be preferred to the CR1 estimator.^63^

Both the CR1 and CR2 adjustments result in the use of a t-distribution for hypothesis testing. For the CR1 approach, however, these degrees of freedom are based entirely on the number of clusters/studies and are the same for all regression coefficients. The CR2 degrees of freedom, however, are estimated using a Satterthwaite approximation.^64^ Tipton^9^ shows that, in general, CR2 has a valid Type I error when the Satterthwaite degrees of freedom are greater than 4. When they are smaller than 4, the approximation can lead to inflated Type I errors. In this case, it is suggested to use a higher standard of evidence (e.g., p < .01 instead of p < .05).

Importantly, Satterthwaite degrees of freedom are a complex function of the number of studies, the variation in the number of estimates per study, and the distribution of the covariate being tested. As a result, in meta-regression, different coefficients can have different associated degrees of freedom. Because of the complexity of the calculation, even when the number of studies is large (e.g., 70), it is possible for these degrees of freedom to be very small, and it is not possible to know—without calculating them—when such small degrees of freedom might occur. It is for this reason that Tipton, Pustejovsky, and Ahmadi^38^ propose that the CR2 method should be the default when estimating cluster robust standard errors.

Our study analyzed how meta-analyses addressed dependence when it arose. To do so, we first recorded if there were dependent effect sizes by noting when the number of effect sizes was larger than the number of studies. If there was dependence (number of effect sizes > number of studies), we then coded how it was addressed. We coded several common approaches: (1) dependence (incorrectly) ignored; (2) a single effect size selected for analysis in each study to induce independence; (3) effect sizes averaged to the study level to remove dependence; (4) use of multilevel modelling (MLM); (5) use of multivariate meta-analysis (MVMA); (6) use of CR1; and (7) use of CR2.

Note that meta-analysts could use a combination of these methods (e.g., MLM and CRSE). Importantly, while we code the methods used in these meta-analyses, we did not assess if these methods fully accounted for dependence. For example, a study with correlated effects might have handled this appropriately through the use of (2) selection, (3) averaging, or (7) CRSE, but might have inappropriately handled this through (4) use of an MLM model (which adjusts for a different type of dependence). However, regardless of approach, we consider any of these methods better than (1) incorrectly ignoring the dependence and proceeding as if the data were independent (e.g., using a conventional random effects model).

Table 15 presents the results. Overall, 57% (571/1000) of the meta-analyses included dependent effect sizes (i.e., number of effect sizes > number of studies). Dependent effects were most common in Business and Economics (84%), Environmental Science (78%), Psychology (77%), and Education (69%). However, of the 571 meta-analyses that had dependent effects, fewer than half (47%) adjusted for this dependence in their analysis. In other words, 53% of meta-analyses with dependent effects treated the data as independent.

**Table 15:** Meta-analyses that account for dependencies

	No. with dependencies	% (no.) addressing	Of (2), % select	Of (2), % avg.	Of (2), % MLM	Of (2), % MVMA	Of (2, % CR1	Of (2), % CR2
Discipline	1	2	3	4	5	6	7	8
Anatomy and Physiology	46	43% *(20)*	20%	35%	25%	0%	5%	25%
Biology	48	44% *(21)*	19%	14%	76%	10%	5%	0%
Business and Economics	84	73% *(61)*	7%	21%	39%	7%	39%	7%
Education	69	67% *(46)*	7%	35%	24%	2%	2%	48%
Engineering	50	22% *(11)*	36%	45%	9%	9%	0%	18%
Environmental Sciences	78	40% *(31)*	42%	23%	52%	0%	0%	0%
Medicine	33	15%*(5)*	20%	0%	80%	0%	0%	0%
Pharmacy, Therapeutics and Pharmacology	39	10%*(4)*	50%	25%	25%	0%	0%	0%
Psychology	77	77% *(59)*	31%	42%	34%	0%	2%	15%
Public Health	47	23% *(11)*	27%	36%	36%	0%	9%	0%

**Overall**	**571**	**47%** ***(269)***	**21%**	**30%**	**38%**	**3%**	**11%**	**16%**

*Note*: Column 1 reports the number of meta-analyses in each discipline that have more estimates than studies, generating a dependency between estimates. The top number in Column 2 reports the percent of meta-analyses that have a dependency that address it using one or more of the following approaches: (i) selecting a subset of estimates from the primary studies, (ii) averaging estimates from the primary studies, (iii) using a multilevel estimator (MLM), (iv) using a multivariate estimator (MVMA), (v) using a CR1 clustered robust estimator of the standard error, and (vi) using a CR2 estimator. The italicized number below the top number in Column 2 is the number of meta-analyses that addressed dependency. Columns 3 through 8 report conditional probabilities. That is, conditional on addressing dependency, what percent of meta-analyses used that particular approach (e.g., selection, averaging, etc.). For example, 46 meta-analyses in Anatomy and Physiology have more estimates than studies. Forty-three percent of these (or 20 of the 46 meta-analyses), address this in some way. Of those 20 meta-analyses, 20% selected a subset of the estimates from the primary studies, 35% averaged some estimates from the primary studies, 25% used a multilevel estimator such as the three-level estimator to estimate the model, no studies used a multivariate estimator, 5% adjusted standard errors using the CR1 estimator, and 25% used the CR2 estimator. Notice that the sum of individual percentages is greater than 100% because some meta-analyses used more than one of these methods to address dependency in their datasets.

Some fields are adjusted for dependence more often than others. For example, Psychology (77%) and Business and Economics (73%) were more likely to adjust for dependence, while Pharmacy, Therapeutics, and Pharmacology (10%) and Medicine (15%) were least likely.

Table 15 also summarizes the methods most commonly used to adjust for dependence. Across fields, effect selection (21%) and effect averaging (30%) were the most frequent approaches. Multilevel models dominated in Biology (76%) and Medicine (80%), while multivariate models were rare, with an overall usage rate of 3%. CRSE methods were most common in Business and Economics and Education, though with markedly different usage patterns for CR1 and CR2. In Business and Economics, CR1 and CR2 accounted for 39% and 7% of cases, respectively; in Education, the corresponding rates were 2% and 48%.

One possible reason dependency is often unaddressed is that, even when studies report multiple estimates, the number of estimates per study may be relatively small (cf. Table 2). However, this can only be a partial explanation. As shown in Appendix C, the proportion of meta-analyses addressing dependency generally stabilized between 60% and 70% once the number of estimates per study exceeded three. Notably, even among meta-analyses with more than 11 estimates per study, approximately one-third still treated the estimates as independent. While the number of estimates per study is an imperfect proxy for dependency, the evidence suggests that many meta-analyses could be improved by explicitly modeling the underlying dependence structure. In light of these observations, we provide the following recommendations:

**Recommendation #4a:** *Meta-analyses should not ignore dependent effects. The nature of dependencies should be explicitly identified and appropriately addressed. Methods should be chosen that are appropriate for the type of dependence, available information, and number of studies.*

**Recommendation #4b:** *Meta-analysts using cluster robust standard errors should estimate CR2 standard errors and degrees of freedom. When Satterthwaite degrees of freedom are smaller than 4, higher standards of evidence should be implemented (e.g., considering a Type I error of 0.01 instead of 0.05 for null hypothesis rejection). CR1 should rarely, if ever, be used because the CR2 estimator generally produces results as good or better.*

### 4.5 Evaluation of disciplines against recommendations

Table 16 concludes this study by evaluating each of the 10 disciplines against our nine recommendations (1a, 1b, 2a, 2b, 3a, 3b, 3c, 4a, and 4b). We classify disciplines into three groups according to their adherence to these recommendations. A discipline is considered to show high compliance when 67% or more of its meta-analyses conform to the recommendation, medium compliance when 34–66% conform, and low compliance when 33% or fewer conform. Blue, yellow, and rose/light red indicate high, medium, and low compliance, respectively. The sources for the compliance rates corresponding to each recommendation are provided in the notes below the table.

**Table 16:** Evaluation of disciplines against recommendations

	Recommendation^a^								
	R1a	R1b	R2a	R2b	R3a	R3b	R3c	R4a	R4b
Discipline	1	2	3	4	5	6	7	8	9
Education	81%	40%	60%	0%	39%	0%	15%	67%	96%
Business and Economics	76%	79%	58%	6%	49%	5%	20%	73%	14%
Psychology	77%	14%	38%	0%	51%	2%	13%	77%	90%
Anatomy and Physiology	47%	21%	NA	NA	31%	0%	2%	43%	83%
Engineering	35%	31%	0%	0%	22%	0%	0%	22%	100%
Public Health	31%	29%	67%	0%	26%	0%	1%	23%	0%
Biology	49%	27%	0%	0%	23%	0%	4%	44%	0%
Environmental Sciences	42%	24%	33%	0%	16%	0%	5%	40%	NA
Medicine	21%	29%	NA	NA	26%	0%	2%	15%	NA
Pharmacy, Therapeutics and Pharmacology	31%	32%	NA	NA	7%	0%	0%	10%	NA

Color categories: Cells are color-shaded to indicate how frequently the disciplines meet the respective recommendations. Blue = High compliance (67% and up). Yellow = Medium compliance (34–66%). Rose/light red = Low compliance (33% and below). Gray = Not applicable (NA). *Note*: The disciplines are arranged in lexicographic order of compliance, beginning with those that meet the largest number of recommendations at the highest threshold (blue cells), followed next by the number of medium-compliance instances (yellow cells), and finally by the number of low-compliance instances (rose cells). ^a^ The recommendations are summarized below with sources for the numbers in the table. Full text of the recommendations is available in Section 4. R1a: Meta-analysts should increase their use of meta-regression to explore sources of effect heterogeneity (*Source*: Column 1 of Table 12). R1b: Analysts should go beyond the use of single variable meta-regression and include multiple covariates where possible (*Source*: Column 2 of Table 12). R2a: Meta-analyses need to ensure that weights are not a function of the effect size estimates (*Source*: Calculated from Columns 2 and 3, Table 13). R2b: Researchers using correlations as effect sizes and testing for publication bias should avoid relying on the correlation’s standard error (COURSE: Calculated from Columns 4–6 of Table 13). R3a: Meta-analyses should correct for publication bias whether or not tests indicate its presence (*Source*: Calculated from Columns 3 and 4 of Table 14). R3b: Selection models and multivariate regression models should be used as robustness checks when correcting publication selection (*Source*: Column 7 of Table 10). R3c: Researchers should select two or more different approaches when correcting for publication bias (*Source*: Calculated from Column 9 of Table 10). R4a: Meta-analyses should not ignore dependent effects (*Source*: Column 2 of Table 15). R4b: Meta-analysts using cluster robust standard errors should use CR2 standard errors and Satterthwaite degrees of freedom rather than CR1 standard errors. (*Source*: Calculated from Columns 7 and 8 of Table 15). NA: Indicates that there were no observations that met the condition(s) of the recommendation.

Disciplines are arranged in the table in lexicographic order of compliance, beginning with those that satisfy the largest number of recommendations at the highest threshold (blue cells), followed by the number of medium-compliance instances (yellow cells), and finally the number of low-compliance instances (rose cells). Individual compliance rates can be misleading. For example, the table reports that 0% of Biology meta-analyses complied with Recommendations #2a and #2b, but this is based on only a single meta-analysis in that discipline (cf. Column 1, Table 13). Nevertheless, while no single figure should be overinterpreted, the overall pattern is clear: despite widespread availability of guidelines and best-practice publications, most disciplines fall short in following them.

## 5 Conclusion

This study provides the most comprehensive cross-disciplinary snapshot to date of meta-analytic practice, drawing on evidence from 1,000 studies across 10 diverse fields. The findings reveal substantial variation in study size, types of effect sizes, statistical methods, and the handling of key methodological issues such as publication bias, heterogeneity, and statistical dependence. Beyond documenting differences across disciplines, we identify notable gaps between current practice and established best-practice guidelines.

To address these gaps, we recommend: (1) wider and more sophisticated use of meta-regression to explain heterogeneity; (2) better handling of correlations as effect sizes, including transformations or alternative weighting, and avoiding bias-prone tests; (3) routine correction for publication bias, using multiple complementary methods; and (4) explicit treatment of dependent effect sizes through multilevel, multivariate, or CR2-based robust variance estimation.

We hope this study will serve as a resource for researchers conducting their first meta-analyses, a benchmark for simulation study design, and a reference for applied meta-analysts seeking to align their methods more closely with best-practice standards.

## Data Availability

The data and programming code used to produce the tables and figures in this paper are available at https://osf.io/6dgpn/. All results should be push-button replicable.

## Footnotes

1 See https://replicationnetwork.com/2021/05/18/duan-reed-how-are-meta-analyses-different-across-disciplines/

2 For more on Summon, see https://exlibrisgroup.com/products/summon-library-discovery/.

3 Note that the median does not have to be a whole number or integer. Say that 100 meta-analyses are rank ordered from smallest to largest number of estimates per study. If the median meta-analysis had 11 estimates from 10 studies, the “median number of estimates per study” would be 1.1.

4 We separated Fisher’s *z* from correlations due to several important differences. Although conceptually related, correlations are bounded between −1 and 1 and are directly interpretable, whereas Fisher’s *z* is unbounded and not intuitively interpretable in the same way. Most importantly, their standard errors differ: the standard error of *r* depends on the correlation itself, while that of Fisher’s *z* depends only on the degrees of freedom.

## Appendix

**Appendix A:** Percent of meta-analyses testing and finding publication bias: Comparison of experimental and observational meta-analyses

		Testing for PB		Finding PB	
	Pct. experimental	Exp.	Observ.	Exp.	Observ.
Discipline	1	2	3	4	5
Education	48%	87.5%	82.7%	33.3%	25.6%
Pharmacy, Therapeutics and Pharmacology	41%	73.2%	74.6%	16.7%	15.9%
Engineering	35%	48.6%	63.1%	29.4%	12.2%
Anatomy and Physiology	31%	83.9%	79.7%	38.5%	36.4%
Environmental Sciences	30%	46.7%	62.9%	14.3%	27.3%
Psychology	29%	93.1%	88.7%	59.3%	28.6%
Biology	26%	80.8%	73.0%	33.3%	35.2%
Public Health	24%	54.2%	73.7%	30.8%	35.7%
Medicine	18%	33.3%	67.1%	0.0%	34.5%
Business and Economics	8%	50.0%	76.1%	75.0%	41.4%

**Overall**	**29%**	**69.0%**	**73.9%**	**33.0%**	**30.5%**

*Note*: The values in Column 1 of the table are based on 100 meta-analyses for each discipline, so the percentages in that column also provide the counts (e.g., 48% = 48 meta-analyses in Education synthesize results from experimental studies). The values in Columns 2 and 3 report conditional probabilities. For example, 87.5% of the 48 experimental meta-analyses in Education test for publication bias. 82.7% of the 52 observational meta-analyses in Education test for publication bias. The values in Columns 4 and 5 are interpreted similarly. 33.3% of the experimental meta-analyses in Education that tested for publication bias (25.6% of the observational meta-analyses that tested for publication bias) concluded there was publication bias.

## Appendix

**Appendix B:** Assessing the scope for meta-regression in studies that currently do not conduct a meta-regression or only use a single predictor

	No meta-regression			Single-covariate meta-regression		
		% ≥ 40	% ≥ 50		% ≥ 40	% ≥ 50
	Count	estimates	estimates	Count	estimates	estimates
Discipline	1	2	3	4	5	6
Anatomy and Physiology	53	8%	6%	37	27%	24%
Biology	51	18%	12%	36	64%	56%
Business and Economics	24	54%	46%	16	69%	63%
Education	19	16%	11%	49	47%	43%
Engineering	65	22%	22%	24	42%	33%
Environmental Sciences	58	38%	33%	32	84%	81%
Medicine	79	9%	6%	15	33%	13%
Pharmacy, Therapeutics and Pharmacology	69	10%	4%	21	24%	10%
Psychology	23	35%	30%	66	59%	56%
Public Health	69	16%	12%	22	32%	23%

**Overall**	**510**	**19%**	**15%**	**318**	**50%**	**44%**

*Note*: This table examines two subsets of meta-analyses: (i) those that did not conduct a meta-regression, and (ii) those that conducted a meta-regression using only one explanatory variable. For each subset, the table reports the number of meta-analyses, along with the percentage that included 40 or more estimates and 50 or more estimates. For example, among the 100 meta-analyses in Anatomy and Physiology, 53 did not conduct a meta-regression. Of these, 8% had 40 or more estimates, and 6% had 50 or more, suggesting that these studies may have had sufficient data to explore heterogeneity through meta-regression. Conversely, 37 of the 100 studies conducted a meta-regression with only one explanatory variable. Among them, 27% had 40 or more estimates, and 24% had 50 or more, indicating that some may have had enough data to support a more complex analysis with multiple explanatory variables.

## Appendix

**Appendix C:** Relationship between estimates/study and the percent of meta-analyses addressing dependency

Estimates/ study	No. with dependencies	No. addressing	% addressing	Of (2), % select	Of (2), % avg.	Of (2), % MLM	Of (2), % MVMA	Of (2), % CR1	Of (2), % CR2
	1	2	3	4	5	6	7	8	9
1	429	0	–	–	–	–	–	–	
> 1 to 2	308	113	37%	32%	51%	19%	1%	2%	12%
> 2 to 3	70	38	54%	21%	29%	42%	5%	3%	21%
> 3 to 4	39	25	64%	16%	16%	52%	4%	4%	28%
> 4 to 5	29	14	48%	21%	7%	64%	0%	0%	21%
> 5 to 6	20	12	60%	8%	17%	67%	0%	17%	8%
> 6 to 7	21	13	62%	15%	15%	62%	0%	8%	31%
> 7 to 9	20	12	60%	8%	17%	67%	8%	8%	8%
> 9 to 11	15	9	60%	0%	0%	67%	11%	33%	11%
> 11	49	33	67%	3%	3%	36%	6%	55%	12%

**Overall**	**1,000**	**269**	**47%**	**21%**	**30%**	**38%**	**3%**	**11%**	**16%**

*Note*: This table is similar to Table 15 except that it groups meta-analyses by the number of estimates per study rather than by discipline. Column 1 reports the number of meta-analyses in each category. Column 2 reports the number of meta-analyses that in some way address the dependency that arises from having more than one estimate per study. There are a total of 571 meta-analyses with estimates/study >1 (571 = 1,000–429). Column 3 reports the percent of these that address dependency, both by category and overall. So, for example, the overall “% Addressing” is 269/571 = 47%. Columns 4–9 report the percent of meta-analyses that have a dependency and address it using one or more of the following approaches: (i) selecting a subset of estimates from the primary studies, (ii) averaging estimates from the primary studies, (iii) using a multilevel estimator (MLM), (iv) using a multivariate estimator (MVMA), (v) using a CR1 clustered robust estimator of the standard error, and (vi) using a CR2 estimator. Columns 4–9 report conditional probabilities. That is, conditional on addressing dependency, what percent of meta-analyses used the given approach.
